# Biomaterials and advanced technologies for the evaluation and treatment of ovarian aging

**DOI:** 10.1186/s12951-022-01566-8

**Published:** 2022-08-11

**Authors:** Meng Wu, Yican Guo, Simin Wei, Liru Xue, Weicheng Tang, Dan Chen, Jiaqiang Xiong, Yibao Huang, Fangfang Fu, Chuqing Wu, Ying Chen, Su Zhou, Jinjin Zhang, Yan Li, Wenwen Wang, Jun Dai, Shixuan Wang

**Affiliations:** 1 Department of Obstetrics and Gynecology, Tongji Hospital, Tongji Medical College, Huazhong University of Science and Technology, Wuhan, 430030 Hubei China; 2 National Clinical Research Center for Obstetrical and Gynecological Diseases, Wuhan, 430030 Hubei China; 3 Key Laboratory of Cancer Invasion and Metastasis, Ministry of Education, Wuhan, 430030 Hubei China; 4 Department of Obstetrics and Gynecology, Zhongnan Hospital of Wuhan University, Wuhan, 430071 Hubei China

**Keywords:** Ovarian aging, Biomaterials, Evaluation, Treatment

## Abstract

Ovarian aging is characterized by a progressive decline in ovarian function. With the increase in life expectancy worldwide, ovarian aging has gradually become a key health problem among women. Over the years, various strategies have been developed to preserve fertility in women, while there are currently no clinical treatments to delay ovarian aging. Recently, advances in biomaterials and technologies, such as three-dimensional (3D) printing and microfluidics for the encapsulation of follicles and nanoparticles as delivery systems for drugs, have shown potential to be translational strategies for ovarian aging. This review introduces the research progress on the mechanisms underlying ovarian aging, and summarizes the current state of biomaterials in the evaluation and treatment of ovarian aging, including safety, potential applications, future directions and difficulties in translation.

**Graphical Abstract:**

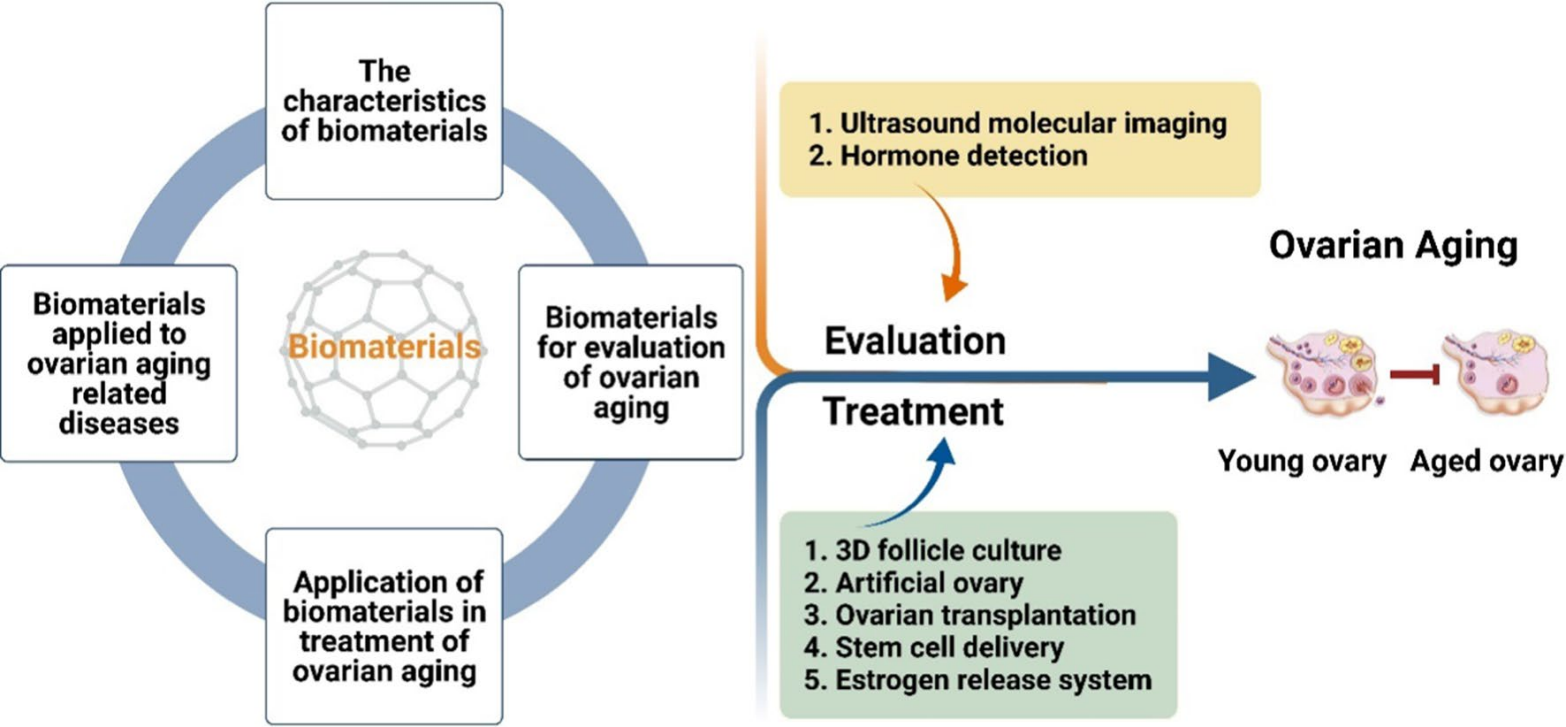

## Introduction

Ovarian aging is characterized by a progressive decline of ovarian function, manifested by a decrease in the quantity and quality of oocytes with advancing age. The ovary is one of the first organ systems to show hallmarks of aging, in comparison to other organs. Most countries show an increasing number of women’s first pregnancies at what is considered an advanced reproductive age (≥ 35 years). With advancing age, difficulty in conceiving and infertility increased. Similarly, oocytes derived from women of advanced age have higher chance of resulting in miscarriage, and/or aneuploid offspring [1]. The end point of ovarian aging is menopause, most women experience menopause around the age of 50 years [2]. The average life expectancy of women has increased to more than 78 years that means nearly a third of a woman's life will be spent after menopause, accompanied by hot flashes, night sweats, irritability, depression, and other menopausal syndrome. Importantly, ovarian aging drives the aging of multiple organs, which is considered as the pacemaker of female body aging [3]. Ovarian aging can lead to obesity, diabetes, Alzheimer's disease, urogenital atrophy, osteoporosis and fracture, cardiovascular disease, and an increased all-cause mortality, which seriously decrease the life quality of aged female [4, 5]. Therefore, the treatment strategies that can delay ovarian aging would improve fertility and health in females.

Over the last two decades, some therapeutic strategies to improve, reverse or slow ovarian aging have emerged. Hormone replacement therapy (HRT) is a universal treatment for ovarian aging, which could allow women to free themselves from the malediction of menopause and conserve their fertility [6]. However the use of HRT has been vigorously debated [7], previous studies revealed that HRT was associated with an increased risk of venous thromboembolism [8], cancer risk [9], and ischemic stroke [10]. In recent years, interest has rapidly grown in studies exploring the therapeutic potential of stem cells in ovarian aging. Different types of stem cells, including embryonic stem cells (ESCs), mesenchymal stem cells (MSCs), stem cells from extraembryonic tissues, induced pluripotent stem cells (iPSCs) and ovarian stem cells [11], have therapeutic effects on ovarian damage. However, transplantation rejection, tumorigenicity, genetic instability and ethical issues with stem cells limited their use [12–14]. Furthermore, some other methods, such as mitochondrial therapy, antioxidants, epigenetic regulators, telomerase activators and traditional Chinese medicine, have been used to prevent ovarian aging, while clinical trials have not yet been conducted on most of these therapies. Therefore, advanced therapeutic strategies to delay, or partially reverse symptoms of ovarian aging are urgently needed.

Biomaterials have the advantages of promoting cell interactions, good passive and active targeting, good stability and biodegradability, high drug loading content and controlled drug release [15–20]. For decades, a large number of studies have focused on evaluating the potential of biomaterials for various applications including regenerative medicine and anti-aging. For example, in age-related macular degeneration (AMD), Suri et al. for the first time delivered chitosan modified poly (lactic-co-glycolic acid) (PLGA) nanoparticles containing sirolimus to the posterior segment of the eye via the subconjunctival route for the treatment of AMD in rat models, achieving slow degradation and the necessary long-term sustained drug release while minimizing systemic exposure [21]. In addition, in age-related brain diseases, Chang et al. constructed electrically magnetized gold nanoparticles (AuNPs) to improve cognitive function and memory consolidation by promoting adult hippocampal neurogenesis [22]. Based on the advantages and significant effects of biomaterials in the field of antiaging, the application potential and value of biomaterials for the management of ovarian aging have been gradually recognized by researchers.

In this review, we focus on the research progress on the potential mechanisms of ovarian aging and summarize the current state of biomaterials in the diagnosis and treatment of ovarian aging, including safety, potential applications, future directions and the difficulties in translation, which could help to provide support and guidance for future scientific research and clinical applications.

## Ovarian aging

### Factors of ovarian aging

**Fig. 1:**
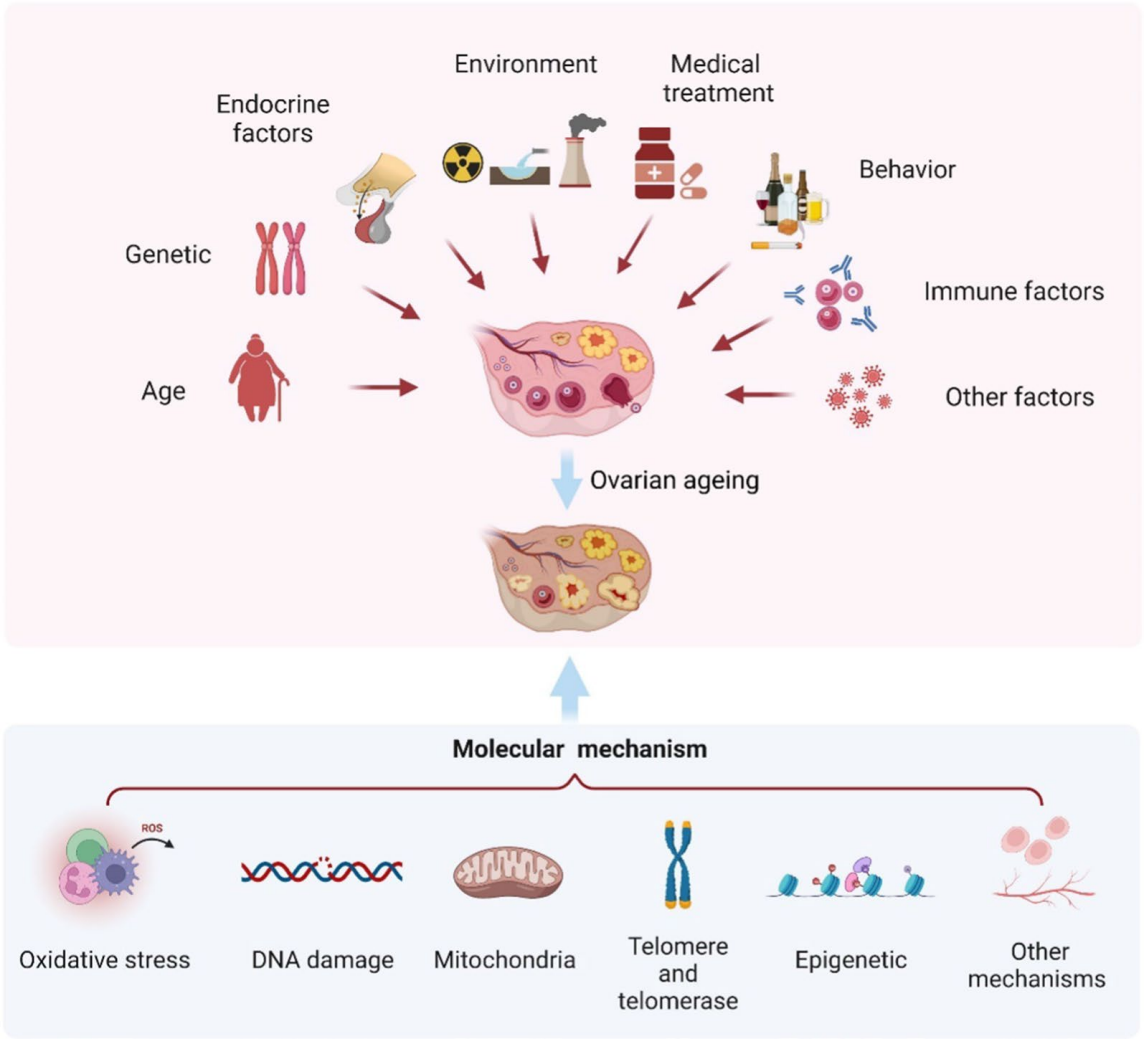
The factors and the molecular mechanisms of ovarian aging

Ovarian aging is a complex process of multifactor and multilink interactions, and the etiology of ovarian aging has not yet been fully elucidated. The main factors of ovarian aging include age, genetics, the hypothalamus and pituitary glands, environment, medical treatments, behaviors, infection, immunity, the endocrine system, and social psychology (Fig. 1).

#### Age

The number of follicles decreases with the increasing age. After 38 years of age, the number of follicles is rapidly consumed, and they number fewer than 1000 in the ovary at the time of menopause [23]. The same is observed for the quality of follicles. From the age of 38, the follicle quality declines rapidly, leading to greatly reduced pregnancy and live birth rates. Therefore, the number and quality of follicles are closely related to age, and age is one of the most important factors in ovarian aging.

#### Genetics

Genetic causes account for approximately 20% to 25% of patients with premature ovarian failure (POF). POF shows a high degree of heterogeneity in genetic variation, including abnormalities in chromosome number and structure, chromosome fragment abnormalities, and single-gene perturbations [24]. The mutated genes associated with ovarian aging are mainly related to the processes of oocyte meiosis, follicle development, hormone synthesis and secretion, DNA damage and repair, and mitochondrial function [25, 26]. However, the related genes known currently can only explain 15% of the genetic causes of ovarian aging [27]. Therefore, the application of clinical orientations for genetic testing is needed for the evaluation of ovarian aging.

#### Environment

A large number of epidemiological investigations have shown that environmental factors can adversely affect primordial follicle establishment, oocyte meiosis, follicle formation, steroid hormone synthesis and fertility, which are associated with decreased ovarian reserve [28]. For example, high concentrations of PM2.5 in the air, polycyclic aromatic hydrocarbons (PAHs) in cigarette smoke and automobile exhaust, heavy metals (lead, mercury, cadmium) in polluted water sources, pesticides remaining in fruits and vegetables, plastic components in packaging bags and other possible environmental factors can affect the reproductive health of female mammals, suggesting that such exposures can lead to premature ovarian aging (POA) in women [29]. However, more experimental investigations in humans are needed to identify their direct and indirect effects on the ovary function, and to characterize their mechanisms of action.

#### Medical treatment

In the process of clinical treatment, many medically related factors such as chemotherapy drugs, radiotherapy and surgical injury, can damage ovarian function. The adverse effects of chemotherapy, radiotherapy and surgery on ovarian function have long been recognized, and there have been increasingly detailed data documenting the effects on short-term markers of ovarian function, longer-term fertility and risk of early menopause [30–32]. The last decade has seen the development of a number of potential methods for protecting the ovaries against damage from chemotherapy or radiotherapy. However, most of that work has been performed using animal models, and it is worth exploring how to minimize the risk of ovarian damage with inevitable medical injury.

#### Behaviors

Poor living habits and behaviors also have adverse effects on ovarian function. A meta-analysis suggested that smoking is associated with a decreased age of menopause of 0.90 years (95% CI 1.58–0.21) [33]. Evidence on the impact of alcohol consumption on female fertility has been quite inconsistent, although the majority of studies have suggested that drinking alcohol damages to ovarian function [34]; nevertheless, moderate alcohol consumption might be unrelated to female fertility [35]. Moreover, both smoking and alcohol consumption might lead to epigenetic changes and DNA damage in germ cells, potentially resulting in inherited imprinting and genetic defects [36].

#### Endocrine factors

The endocrine system maintains and regulates various complicated vital life activities by secreting hormones. The ovary, together with two major neuroendocrine organs, the hypothalamus and the pituitary gland, constitutes the hypothalamic pituitary ovarian (HPO) axis, which is considered to be a classical circuit regulating the female reproductive endocrine system. Abnormal function and endocrine organ diseases, such as thyroid disease and diabetes, affect ovarian function via direct and indirect interactions with the HPO axis. Pooling the results of several studies that have investigated the prevalence of autoimmune thyroid disease (AITD) in women with infertility demonstrated a significantly increased incidence of AITD compared to controls, with an overall estimated relative risk of 2.1 (P < 0.0001) [37]. Hypothyroidism can impair pulsatile secretion of gonadotropin-releasing hormone (GnRH), resulting in ovulatory dysfunction and insufficient corpus luteum development [38]. In patients with diabetes mellitus, a hyperglycemic environment promotes neuronal apoptosis, leading to disordered HPO axis secretion [39]. Additionally, diabetes can also directly cause follicle dysfunction [40]. Although there have been many studies of the correlation between the endocrine system and ovarian aging, the underlying mechanism has yet to be fully elucidated.

#### Immune factors

It is well known that immune factors play a crucial role in ovarian aging. Studies have shown that autoimmune abnormalities account for 10% to 30% of premature ovarian insufficiency (POI), including anti-ovarian autoantibodies, immune oophoritis, thyroiditis and rheumatoid arthritis [41]. The most abundantly present types of innate immune cells in the ovaries are macrophages. Zhang et al. revealed a significantly M2 polarized and increasingly monocyte-derived macrophage population in the old ovary compared to that in the young ovary [42]. Intriguingly, M2 macrophages are known to deposit collagens in the extracellular matrix (ECM), in turn contributing to the development of fibrosis in the ovary during aging [43]. Furthermore, cytokines are the key substances that mediate immune biological processes. Mechanistically, there is evidence that cytokines can influence oocyte quality, ovarian reserve, ovarian steroid production, and the follicular microenvironment, thereby further contributing to ovarian aging [44–47]. For example, elevated levels of the proinflammatory cytokines interleukin-1 alpha (IL-1α), interferon gamma (IFN-γ), and tumor necrosis factor alpha (TNF-α) have been found in the serum of patients with POI [48]. Therefore, anti-immune inflammation aging therapies will become an important strategy in the prevention and treatment of ovarian aging.

#### Other factors

Infection is also one of the influencing factors of ovarian aging. Studies have demonstrated that bacterial or viral infection can lead to abnormal menstruation, decreased reproductive function, and even amenorrhea or POA [34].

### The molecular mechanisms of ovarian aging

Ovarian aging is essentially a process of gradual depletion of the primordial follicle pool, influenced by the complex regulatory network inside and outside the body, such as DNA damage, epigenetic changes, free radical balance disorders, and abnormal mitochondrial function and so on (Fig. 1).

#### Oxidative stress

Free radicals play an indispensable role in the physiological changes in the ovaries, such as angiogenesis, sex hormone synthesis, ovulation, and formation and dissolution of the luteum [49]. Oxidative stress, caused by the imbalance between the production and destruction of reactive oxygen species (ROS), directly damages the intraovarian environment and many other cells. Excessive ROS induce apoptosis of granulosa cells (GC) and/or oocytes, leading to follicular atresia, directly or indirectly activating primordial follicles, and accelerating the decline of ovarian reserve function [50]. Some studies have shown an increase in ROS and a decrease in antioxidant levels in the oocytes, cumulus cells and follicular fluid of older women [51, 52]. Oxidative damage to the ovaries is generally caused by the propagation of lipid peroxidation cascades, which seriously influencing folliculogenesis, meiosis, and ovulation and eventually leading to ovarian aging.

#### DNA damage

Recent evidence has suggested that the DNA damage accumulates with age, possibly due to reduced DNA repair capacity with age in the oocytes of humans and mice [53]. Katherine et al. revealed that the genes related to the DNA damage response (DDR) process regulate ovarian reserve and its depletion rate and determine the age of natural menopause [54]. With the development of sequencing technology, an increasing number of candidate genes related to DNA damage and repair in ovarian aging have been found, including MCM8, MCM9, MEIOB, MND1, PSMC3IP, HFM1, and MSH5, which affect oogenesis mainly by regulating the process of homologous recombination in meiosis [55–57].

#### Mitochondria

As an important energy-supplying organelle, the mitochondria play a key role in the regulation of calcium homeostasis, oxidative phosphorylation, the cell cycle, senescence and apoptosis. As age-related alterations have been documented in mitochondrial function, the mitochondrial DNA mutation load and mitochondrial DNA copy numbers in mammalian oocytes have been investigated as potential biomarkers of oocyte quality [58]. Women undergoing in vitro fertilization (IVF) who are carriers of mitochondrial DNA mutations demonstrate decreased ovarian reserve based on lower anti-Müllerian hormone (AMH), lower antral follicle count (AFC), and a smaller number of oocytes retrieved than healthy volunteers [59]. Similarly, the mitochondrial DNA copy number is also lower in the unfertilized oocytes from women with infertility problems [60]. Therefore, mitochondria play a key role in ovarian aging.

#### Telomeres and telomerase

Telomeres and telomerase are closely related to aging and apoptosis. In recent years, studies have revealed that changes in telomere length and telomerase activity might be among the important mechanisms of ovarian aging [61]. It has been shown that oocytes in women with advanced age have shorter telomeres than young women, and this difference leads to a higher percentage of miscarriages or aneuploid embryos [62]. Similarly, women with a low pregnancy rate or POI showed shorter telomeres than healthy controls [62, 63]. Uysal et al. suggested that decreased telomerase reverse transcriptase (TERT) and telomere-binding protein expression might underlie the telomere shortening of ovaries with age, which could be associated with female fertility loss [64]. These data together argue that the telomere pathway is critical in ovarian aging.

#### Epigenetics

Epigenetics is considered to be an important cause of ovarian aging. It has been reported that common epigenetic modifications such as DNA methylation, ribonucleic acid (RNA) methylation, histone acetylation, phosphorylation and ubiquitin, could be involved in the occurrence and development of ovarian aging [65]. Kristina et al. reported differential methylation variability between diminished ovarian reserve (DOR) and normal ovarian function, indicating that the unstable methylome in granulosa cells can cause epigenetic dysfunction, resulting in poor ovarian reserve [66]. A study of histone modification showed that phosphorylation of histone H3 regulates the initiation of granulosa cell differentiation [67]. Currently, there are still few studies of epigenetic modification in ovarian aging, and some of the conclusions have been controversial. Therefore, future in-depth studies of epigenetics could have profound implications for ovarian aging.

#### Other mechanisms

The ovarian microenvironment is involved in follicular formation, development, maturation and ovulation [68], imbalances in which will lead to abnormal ovarian function. The accumulation of extracellular matrix, abnormalities in the vascular system, and the accumulation of senescent cells will lead to ovarian microenvironment disorder [69]. In addition, recent studies have demonstrated that there are oogonial stem cells in the ovaries [70], and the loss of stem cell function or instability of stem cell nests leads to ovarian imbalance, resulting in ovarian aging.

## The characteristics of biomaterials

**Fig. 2:**
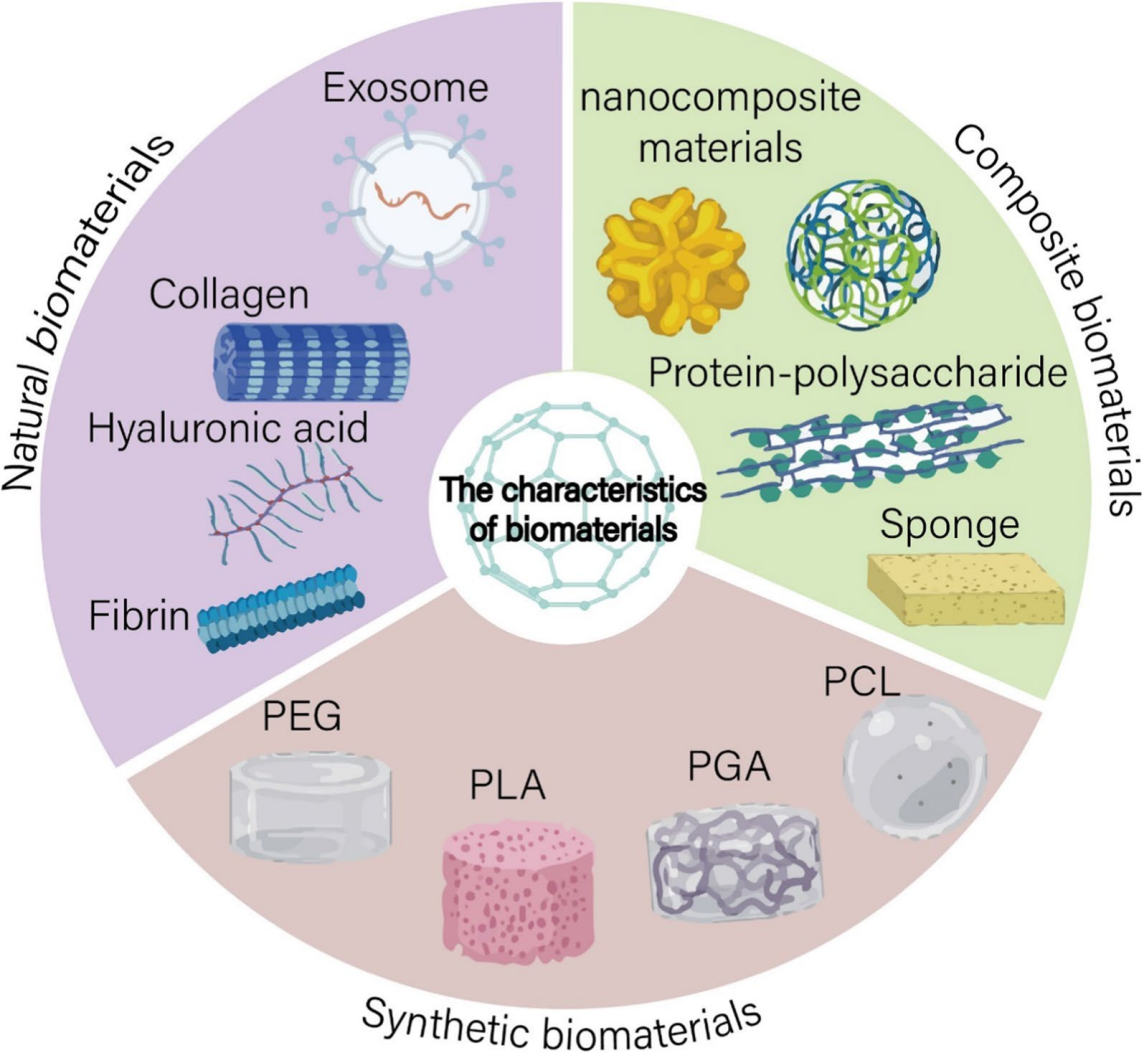
The characteristics of biomaterials

Biomaterials can be classified into three basic categories: natural biomaterials (including extracellular vesicle, collagen, hyaluronic acid, fibrin, etc.), synthetic biomaterials (including polylactic acid (PLA), polyglycolic acid (PGA), polycaprolactone (PCL), polyethylene glycol (PEG), etc.) and composite biomaterials (including protein-polysaccharide composite biomaterials, nanocomposite biomaterials, sponges, etc.) (Fig. 2). They have been designed as theranostics showing unparalleled advantages, such as promoting favorable cellular interaction, relatively high drug loading content, controllable drug release, excellent passive and active targeting, good stability, biodegradability, biocompatibility and low toxicity [15, 16, 71, 72]. Some biomaterials have been utilized for reproductive tissue engineering and regenerative medicine [73, 74]. The characteristics of biomaterials are discussed below, with a focus on those that have been investigated for the tissue engineering.

### Natural biomaterials

Natural biomaterials are well positioned to play a significant role in the development of the next generation of biomaterials for recovery of ovarian function. They are derived from naturally occurring substances and are easily available, have excellent biocompatibility and biodegradability and are well tolerated in vivo. Furthermore, natural polymers contain biomolecules that are natural to the cells, which can support and guide the cells to proliferate and differentiate at particular time interval and consequently can enhance the biological interaction with them [75]. Natural biomaterials can be divided into protein biomaterials (including fibrin, collagen), polysaccharide biomaterials (including hydrogel, alginate, and hyaluronic acid), and natural nanomaterials (extracellular vesicles, EVs). Protein-based biomaterials are flexible in structure and have good biocompatibility [76]. In addition, they have natural cell adhesion sites, making them ideal materials for biomaterials engineering [77]. Polysaccharide biomaterials that can be readily available from plants, animals, bacteria, etc. [78], are therefore inexpensive and non-toxic [79]. In addition, chitosan, sodium alginate and starch are natural sources of many non-conjugated luminescent polymers (NLP), which show great potential for future application in the design and development of luminescent drug carriers [80]. As a natural nanomaterial, EVs are a heterogeneous group of cell-derived membranous structures comprising exosomes and macrovesicles, which originate from the endosomal system or which are shed from the plasma membrane, respectively [81]. EVs perform an important role in cell-to-cell communication and are involved in multiple physiological and pathological processes, so they have good biocompatibility with immune system and low toxicity.

### Synthetic biomaterials

With the further development of science and technology and economy, the application range of synthetic polymer materials is gradually expanding. Synthetic polyesters, including PLA, PGA, PCL and PEG approved by Food and Drug Administration (FDA) are the most widely studied biodegradable polymers in the reproductive tissue engineering and regenerative medicine field. The synthetic materials have the special properties of being able to cross biological barriers or passively target tissues [82] and avoid some drug delivery problems that could not be effectively solved in the past, including overcoming multidrug resistance and penetrating cell barriers that restrict drugs from reaching their intended targets [83]. Compared with natural biomaterials, the physical, chemical, mechanical and biological properties synthetic biomaterials can be modified to suit the needs of material design [84, 85]. In addition, their materials are more plentiful and therefore cheaper, making them cheaper to manufacture. For example, Obireddy et al. have used inexpensive 2-hydroxyethyl starch synthetic biomaterials to produce co-released particles for use in a variety of drugs [86]. However, although the biocompatibility and biodegradability of synthetic biomaterials are well established in almost all cases, have reached similar safety levels to natural compounds, they carry the risk of toxicity and immunogenicity to the host due to their significant difference to native tissue [84].

### Composite biomaterials

The composite biomaterials definition is basically used to refer to new types of materials that are created by using two or more natural or synthetic polymers together. Nanocomposite materials can be categorized due to its polymer including such as polymer based and non-polymer based (inorganic) [87]. A variety of bioactive composites have been investigated over the last three decades as substitute materials for diseased or damaged tissues in the human body [88]. Proteins (including fibrin, collagen, and elastin) and polysaccharides (including chitosan, cellulose, and alginate) are widely used in composite biomaterials [89]. Proteins can provide better biocompatibility, and polysaccharide can provide further thermal stability and antibacterial properties. The combination of the two can be used as better biomaterials in the field of regenerative medicine [90].

## Biomaterials for the evaluation of ovarian aging

Timely diagnosis of ovarian aging has become an urgent need to improve the quality of life of contemporary women. Bioinformatics involves the research, development, or application of computational tools and methods to obtain, store, visualize, and interpret medical or biological data [91, 92]. In addition, machine learning has become an indispensable tool influencing the fields of bioinformatics and medicine. Machine learning automatically learns complex patterns or rules from big data, mainly for data representation and prediction problems [93]. For example, He et al. developed a Python tool, MRMD2.0, to achieve dimensionality reduction during machine learning [94]. Using bioinformatics to construct biomaterials to detect ovarian function markers for dynamic monitoring of ovarian aging is a noninvasive, effective and convenient technology with high sensitivity and specificity. Next, we summarize innovative strategies for the evaluation of ovarian aging with different biomaterials.

### Biomaterials for anti-Müllerian hormone detection

AMH is a dimer glycoprotein that is a member of the transforming growth factor β (TGF-β) family of growth and differentiation factors [95]. AMH is synthesized by granulosa cells of follicles and released into follicular fluid. It enters the blood circulation through the perifollicular vascular network, so it can be measured in peripheral blood [96]. AMH has been implicated as the most valuable marker of ovarian reserve function because it is consistent throughout the menstrual cycle, with no significant variability between menstrual cycle and not affected by short term use of oral contraceptives [97].

Early detection of AMH is an immune cell chemical detection and immunoradiometric analysis technology, focusing on AMH positioning and functional studies in animal tissues, which have been unable to meet the needs of clinical detection diagnosis [98–100]. The first generation of AMH enzyme-linked immunosorbent assay (ELISA) detection technology uses a pair of paired monoclonal antibodies to AMH, and it has begun to meet the needs of clinical detection, but there is no unified detection standard [101], and its results are susceptible to sample storage and repeated freeze–thaw cycles [102]. In addition, there was a large difference in test results between commercial kits [103]. Beckman Coulter established the second-generation AMH ELISA detection technology and unified the detection standard for AMH [104], but the problem of unstable AMH detection results persisted [102]. In addition, the above detection methods indirectly evaluate ovarian function through AMH in the blood, showing limitations in assessing the real ovarian reserve.

Molecular probes are constructed by modifying antibodies or ligands of disease-specific molecules on the surface of biomaterials, and they constitute a noninvasive, effective and convenient detection technology that can achieve early detection and real-time monitoring of diseases at the molecular level [105]. Zhang et al. developed AMH-targeted nanobubbles (NBAMH) by integrating AMH antibodies into the surface of nanobubbles (NBs) [105]. NBAMH showed high affinity for ovarian granulosa cells in vitro, and the ultrasound signal of transplanted ovaries was significantly enhanced compared to that of untargeted NBs. By designing NBAMH as an ovarian tissue-specific molecular probe, Zhang et al. provided a promising noninvasive tool for the study of dynamic monitoring of early ovarian function after ovarian transplantation. Moreover, Mu’s study also devised a nanoscale AMH targeted contrast agent, which could improve the targeted development ability of rat transplanted ovaries and effectively solve the existing problems of micron grade contrast agents, such as their only being for blood pool imaging and their lack of tissue specificity; in contrast, they could facilitate noninvasive evaluation of transplantation of ovarian function to realize the in vivo transplantation of ovarian development and functional evaluation. Furthermore, Liu et al. introduced time-resolved immunochromatographic technology into the detection of AMH using nanoenhanced time-resolved fluorescence microspheres and prepared a quantitative AMH detection strip combined with a time-resolved fluorescence immunoassay, which effectively improved the sensitivity of the platform and the detection effect of the low-value fluorescence signal. The invention realized the simple, rapid and low-cost detection of AMH, with high sensitivity and small differences between batches, and it provided a method for the realization of bedside detection.

### Biomaterials in estrogen detection

Estrogen can be divided into estrone (E1), estradiol (E2) and estriol (E3), among which E2, produced by granulosa cells of the ovarian follicles, is crucial to maintaining female secondary sexual characteristics and reproductive function [106]. E2 levels are commonly assessed during the early follicular phase of the menstrual cycle, it is a simple, inexpensive, and effective screening tool [107]. Basal levels of E2 have been shown to related with ovarian aging, it falls with age throughout a woman's life [108].

At present, radioimmunoassay is the main method to detect estradiol [109]. Although radioimmunoassay has high sensitivity and specificity, it has the disadvantages of short shelf life and radioactive hazard [109]. In recent years, methods for quantitative detection of serum estrogen have emerged. The main products were enzyme-linked immunosorbent assay [110], fluorescence immunoassay [111], chemiluminescence immunoassay [112] and electrochemiluminescence immunoassay [113]. Conventional immunoassay techniques have been under scrutiny for some time with their selectivity, accuracy and precision coming into question [114]. Chromatographic analysis [115] and ultraviolet (UV) detection [116] have also been used for estrogen detection. However, these methods are expensive, complicated, time-consuming and have different levels of assay sensitivity (0.014–0.04 ng/mL) [110].

Recently, the increasing availability of nanoparticles has attracted widespread attention in the determination of estrogen analysis, because of their high surface areas, high activity, and high selectivity. Li et al. developed a method in which gold nanoparticles enhanced chemiluminescence methods for the measurement of estrogens [117]. Based on the advantages of electrochemical techniques, Jin et al. developed choline derivative-modified electrodes for the assay of estrogens [118]. Subsequently, the same team prepared a Pt nanoclusters/multiwalled carbon nanotube electrochemical biosensor, which had high sensitivity and good reproducibility and stability and could be used as a current-type biosensor for routine analysis of total estrogen in serum [119]. In addition, Huang et al. used AuNP-coupled adaptors to enhance the quantitative detection of 17β-estradiol (17β-E2) specificity by ELISA [120]. Additionally, for the purpose of low-cost and sensitive electrochemical detection of 17β-E2, another study used a multiwall carbon nanotube-Nafion modified electrode [121]. Ovarian 17β-E2 is normally converted into E3, which acts preventively against the occurrence of diseases in women, such as cardiovascular complications and osteoporosis [122, 123]. One study by Gomes et al. created a voltametric sensor based on a cobalt-poly(methionine)-modified glassy carbon electrode that did not require sophisticated instruments or any separation steps, allowing for E3 quantification without laborious and time-consuming procedures [124].

### Biomaterials for follicle stimulating hormone detection

Follicle stimulating hormone (FSH) is a heterodimer expressed by the anterior pituitary gonadotropin, composed of two different subunits, α and β [125], mainly plays a role in the regulation of ovarian follicular generation and steroid generation [125], and is an important indicator of clinical detection of ovarian function [126]. Therefore, timely detection of FSH dynamic changes in women is conducive to the evaluation of ovarian function.

In 1953, Steelman and Pohley first proposed an in vivo specific quantitative determination of FSH, namely the rat ovarian weight gain method [127]. However, this method is cumbersome and not suitable for routine clinical studies, and its sensitivity is too low to detect serum FSH level [128]. With the development of technology, immunoassay methods such as ELISA [129], electrochemiluminescence [130] and chemiluminescence [131] have been widely used in clinical detection of FSH. However, these methods have the disadvantages of requiring many samples, long test time, high cost, low sensitivity and large measurement uncertainty [132]. Therefore, it is urgent to develop a fast, economical and simplified FSH detection and analysis method.

**Fig. 3:**
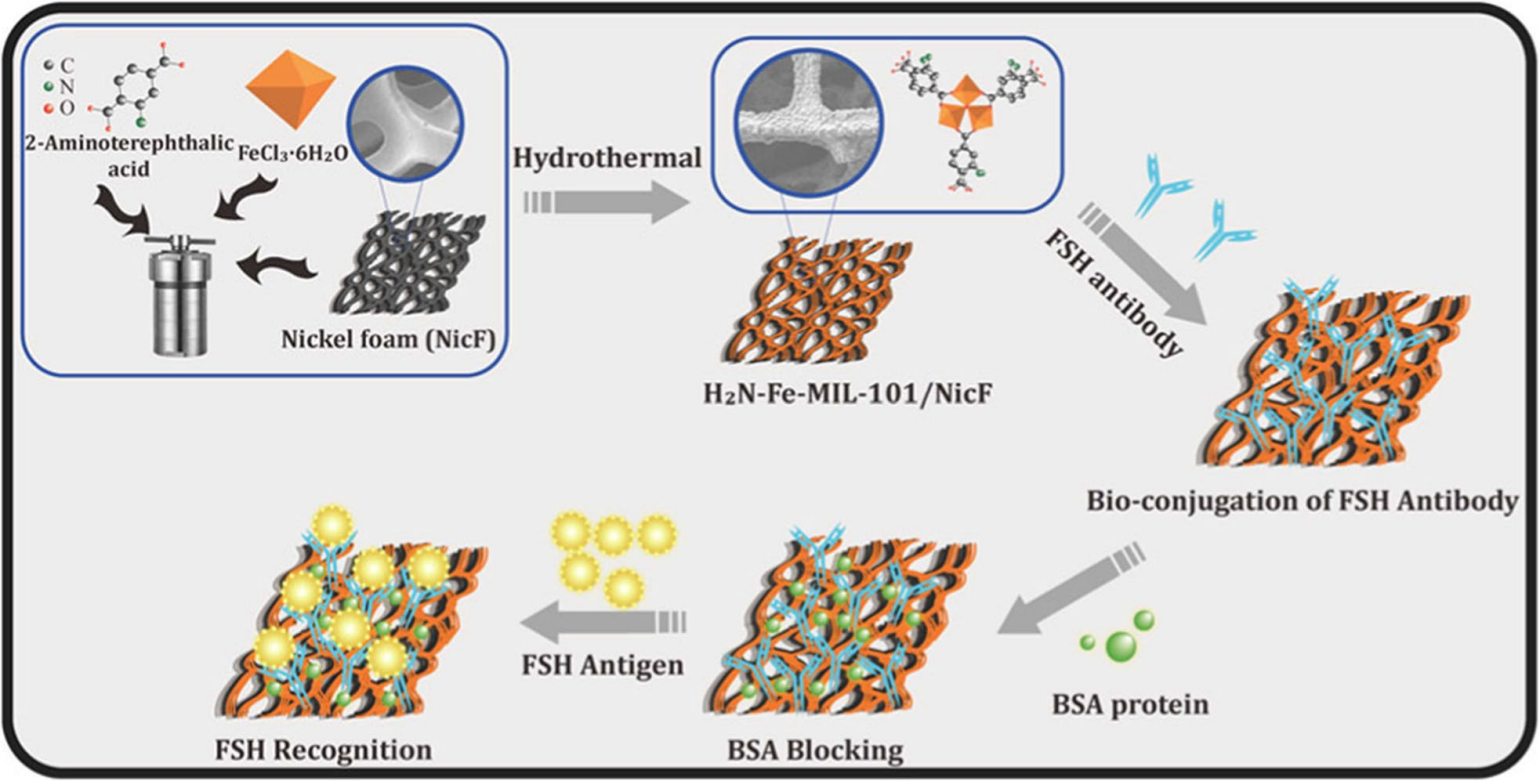
Illustration of the synthesis of iron containing 3D H2N–Fe-MIL-101 nanosheets MOFs on porous NicF substrate by in situ hydrothermal methods derived from FeCl3·6H2O salt and H2Bdc-NH2 ligand precursors and NicF solid support producing uniformly decorated H2N–Fe-MIL-101/NicF electrodes followed by bioconjugation of FSH antibody for FSH detection. (the figure is reproduced from Palanisamy et al. [136] with required copyright permission)

The development of nanotechnology provides new conditions for the development of biomolecular electrical detection systems, but it also provides a new direction for FSH detection [133, 134]. Luo et al. developed a label-free electrochemical immunosensor for the rapid detection of FSH using graphene nanocomposite materials [132]. The method had high sensitivity, fast response and substantial clinical application value. In addition, Lee et al. used a metal-oxide semiconductor silicon nanowire field effect transistor (SINW-FET) device to achieve accurate and rapid detection of FSH [135]. This sensitive, inexpensive, and miniaturized SINW-FET device could serve as an effective sensing method for rapid screening of FSH and menopausal diagnosis. Moreover, Palanisamy et al. synthesized an iron-containing metal–organic framework (H2N –Fe-MIL-101 MOFs) on a porous nickel foam (NicF) substrate by in situ hydrothermal methods, as depicted in Fig. 3. The H2N–Fe-MIL-101/NicF electrode labeled with FSH antibody (Ab-FSH) was applied for specific recognition of an FSH glycoprotein [136]. The material showed fast and excellent sensitivity to FSH. Furthermore, Pareek et al. constructed a novel nanomaterial (NiCo2O4/rGO)-modified indium tin oxide (ITO) electrode for the detection of FSH [137]. The biosensor could help to overcome the disadvantages of current FSH detection methods, such as high cost, long time consumption and low sensitivity, while also providing a dynamic detection range (0.1 pM–1 µM) and a low detection limit (0.1 pM).

### Biomaterials in ovary ultrasound molecular imaging

**Fig. 4:**
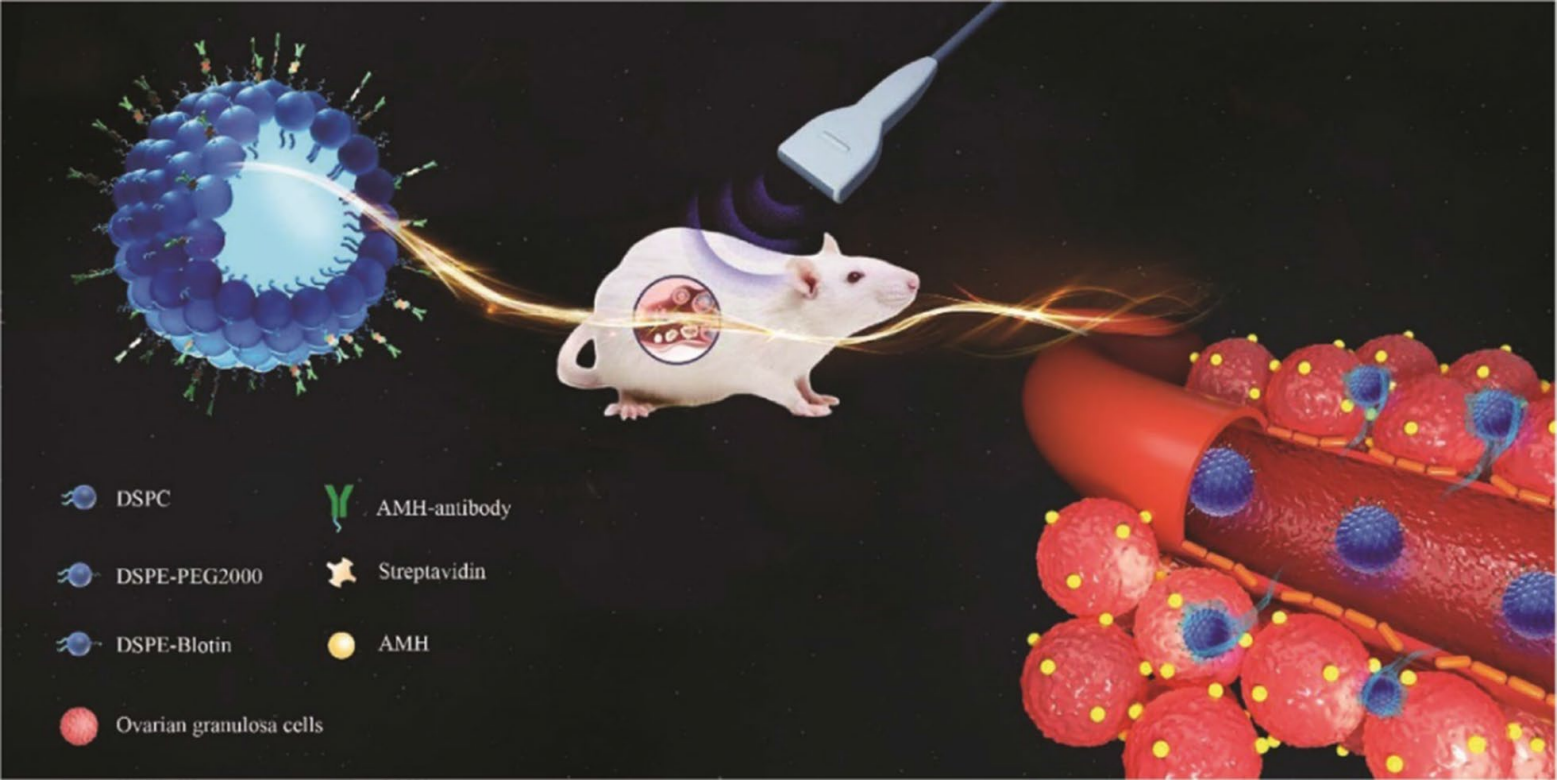
Schematic of AMH-targeted nanobubbles (NBAMH) and their targeting ability to rat ovarian granulosa cells expressing AMH. (the figure is reproduced from Zhang et al. [105] with required copyright permission)

Ultrasonic molecular imaging is a molecular imaging technique based on traditional ultrasound imaging to monitor the level of disease-specific molecular expression [138–140]. With the characteristics of realizing early, noninvasive detection and real-time monitoring of disease at the molecular level [138–140], ultrasonic molecular imaging has great application potential in evaluating follicle survival after early ovarian transplantation. As shown in Fig. 4, Zhang et al. successfully prepared AMH-targeted nanobubbles, which exhibited a high affinity for ovarian granulosa cells in vitro and enhanced ultrasound signals in the ovaries [105]. This new method could be used for early follicle survival detection after ovarian transplantation.

## Biomaterials for ovarian aging therapy

**Fig. 5:**
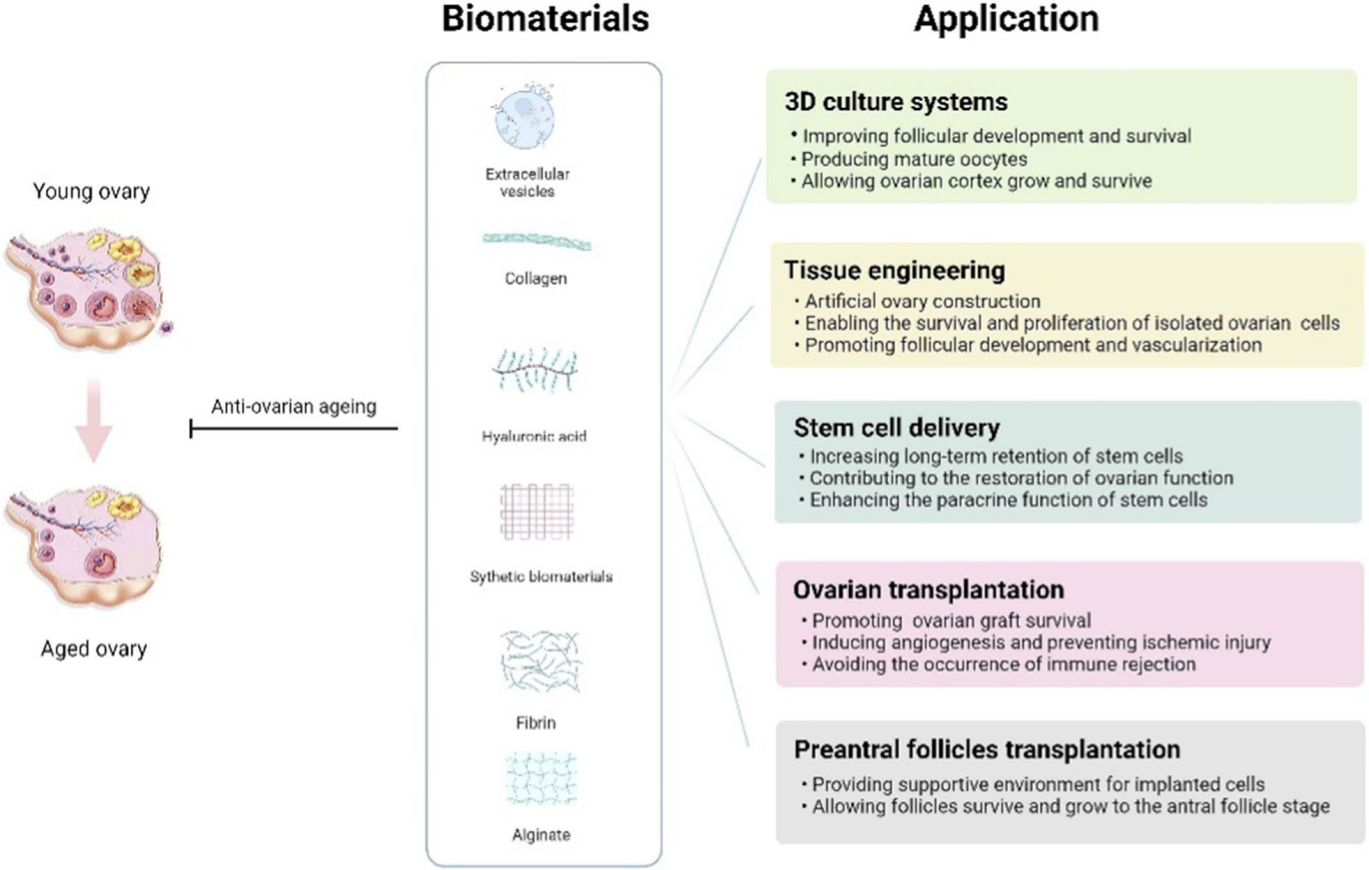
Application of biomaterials in treatment of ovarian aging

Over the last decade, biomaterial technologies have shown great promise as potential treatments for ovarian aging. The ideal biomaterials for ovarian aging therapy should be nontoxic, biocompatible, biodegradable, and bioresorbable. Furthermore, biomaterials should be able to support the regeneration of new cells and tissue without producing an inflammatory reaction [141]. Biomaterials techniques used to treat ovarian aging include the construction of artificial ovaries, systems for the development of follicles, biomaterial encapsulation of cells or drugs, and delivery of natural extracellular vesicles (Fig. 5). Although not yet in the clinical stage, there have been significant developments in this area, including assessments of the effects, safety and feasibility of anti-ovarian aging using biomaterials in animals. The use of these biomaterials and their success in treating ovarian aging (Table 1) and diseases related to ovarian aging are discussed in detail in the following sections.

**Table 1:** List of biomaterials used for the treatment of ovarian aging

Categories	Author	Year	Materials	Model	Major finding	
Extracellular vesicles	Bo Sun [145]	2019	BMSC-derived exosomes	Cisplatin-induced POF mouse model	Inhibited the apoptosis of granulosa cell.	
	Meiling Yang [147]	2020	BMSC-derived exosomes	Cyclophosphamide-induced POF mouse model	Prevented follicular atresia and GCs apoptosis.	
	Zhongkang Li [148]	2021	hUCMSC-derived exosomes	Cyclophosphamide-induced POI mouse model	Reduced cell apoptosis and enhanced proliferation.	
	Conghui Liu [149]	2020	hUCMSC-derived exosomes	Busulfan and cyclophosphamide-induced POI mouse model	Improved the fertility of POI mice without adverse effects on the cognitive behavior of their offspring.	
	Ziling Yang [150]	2019	hUCMSC-derived exosomes	Busulfan and cyclophosphamide-induced POI mouse model	Restored ovarian function by promoting angiogenesis.	
	Chenyue Ding [151]	2020	hUCMSC-derived exosomes	Cyclophosphamide-induced POI mouse model	Reduced ROS levels in the damaged ovary and suppressed SIRT7 expression.	
	Jin Zhang [152]	2020	hUCMSC-derived exosomes	Cisplatin-damaged granulosa cells	Promoted resistance to cisplatin-induced granulosa cells apoptosis and restored synthesis and secretion of steroid hormone in granulosa cells.	
	Liping Sun [153]	2017	hUCMSC-derived exosomes	Cisplatin-damaged granulosa cells	Ameliorated cisplatin-induced granulosa cells stress and apoptosis in vitro.	
	Boxian Huang [155]	2018	hADMSC-derived exosomes	Cyclophosphamide-induced POI mouse model	Inhibited expression of the apoptosis genes in human granulosa cells and improved ovarian function.	
	Chenyue Ding [157]	2020	hAMSCs-derived exosomes	Cyclophosphamide-induced POI mouse model	Improved proliferation, inhibited apoptosis, reduced ROS level and decreased the expression of SIRT4 and relative genes in POI hGCs and ovaries.	
	Qiuwan Zhang [14]	2019	hAECs-derived exosomes	Busulfan and cyclophosphamide-induced POI mouse model	Increased follicles,inhibited GCs apoptosis and protected the ovarian vasculature from damage in POF mice.	
	Guan-Yu Xiao [163]	2016	AFMSCs-derived exosome	Busulfan and cyclophosphamide-induced POF mouse model	Inhibited apoptosis in damaged GCs and prevented ovarian follicles from atresia.	
	Eman Thabet [164]	2020	AFMSCs-derived extracellular vesicles	Cyclophosphamide-induced premature ovarian dysfunction rats model	Restored total follicular counts, AMH levels,regular estrous cycles and fruitful conception.	
	Siwen Zhang [166]	2021	MenSCs-derived exosomes	4-Vinylcyclohexene diepoxide-induced POI mouse model	Promoted follicular development, restored fertility and improved live birth.	
	Chenfeng Yuan [172]	2021	Follicular fluid exosomes	Porcine granulosa cells	Increased the proliferation and progesterone synthesis of porcine ovarian granulosa cells.	
	Samuel Gebremedhn [173]	2020	Follicular fluid exosomes	Bovine granulosa cells	Protected against heat stress by reducing the amount of ROS accumulation.	
	Thais A Rodrigues [174]	2019	Follicular fluid exosomes	Cultured cumulus—oocyte complex	Increased the resistance of the oocyte to heat shock and improved the cleavage and blastocyst rates.	
Extracellular matrix	Monica M Laronda [180]	2015	SDS	Ovariectomized mice	It could significantly change ECM, and had a strong destructive effect on the ultrastructure of natural tissues.	
	S E Pors [186]	2019	0.1% SDS and DNA enzymes	Immunodeficient mice	Adequately decellularized both human ovarian medullary and cortical tissue by eliminating all cells and leaving the ECM intact.	
	Wen-Yue Liu [187]	2017	Triton X-100 solution and DNA enzyme	Rats	Had no cytotoxicity to rat ovarian cells in vitro and only caused minimal immunogenic response in vivo.	
	Maryam Nezhad Sistani [188]	2021	1%Triton X-100 and 0.5%SDS	The endometrial mesenchymal cells	It could effectively decellularize human ovarian tissue and highly preserve ECM content and non-cytotoxic properties.	
	Farideh Eivazkhani [184]	2019	NaOH used as a satisfactory decellularization agent	Ovariectomized mice	It supported follicular reconstruction better than SDS.	
	Ashraf Hassanpour [179]	2018	SLES as an ionic detergent	Ovariectomized rats	Preserved the structure and composition of ovarian ECM, and promoted in vitro and in vivo biocompatibility and neovascularization of biological ovarian scaffides.	
	Hossein Nikniaz [190]	2021	Human and bovine acellular ovarian scaffold	Mouse preantral follicles	Sodium alginate containing acellular ovarian scaffold could maintain follicular viability in vitro.	
	Sanaz Alaee[191]	2021	Decellularized rat ovarian scaffold	Preantral follicles from prepubertal mice	The preantral follicles transformed into antral follicles, and produced mature meiosis oocytes.	
	Wen-Yue Liu[187]	2017	Porcine acellular scaffold	Rat ovarian tissue	Supported the adhesion, migration, and proliferation of immature female rat granulose cells and showed estradiol secretion.	
	S E Pors [186]	2019	Acellular human ovarian tissue	Human preantral follicles	Supported the survival of human follicles.	
	Eun Jung Kim [192]	2020	ECM-derived hydrogel	Mouse ovarian follicles	Supported follicular morphology and growth, and promoted oocyte maturation.	
	Ashraf Hassanpour [179]	2018	Acellular scaffold of human ovarian tissue	Ovariectomized mice	Increased vaginal opening and estrogen levels after implantation and confirmed the onset of puberty.	
	Monica M Laronda [180]	2015	Acellular bovine ovarian scaffold	Ovariectomized mice	Supported the growth of isolated mouse follicles, and produced estrogen and reconstructed menstrual cycles.	
	Georgia Pennarossa [193]	2021	Porcine ovarian 3D biological scaffold	Female germ line stem cells	Represented a powerful tool for in vitro recreation of a bioengineered ovary that might constitute a promising solution for hormone and fertility function restoring.	
	Kutluk Oktay[194]	2016	Human extracellular tissue matrix scaffold	Human	Pregnancies had been reported following minimally invasive transplantation of previously cryopreserved ovarian tissue.	
Collagen	Sunyoung Joo[198]	2016	Collagen-rich, biomimetic 3D shells	Rodent ovarian follicles	Collagen hydrogel properties were important for follicular phenotype and function maintenance.	
	C Torrance [199]	1989	A collagen gel matrix	Mouse preantral follicles	Allowed mouse follicles to separate and grow in vitro for at least 2 weeks.	
	G Taru Sharma [200]	2009	A 3D collagen gel culture system	Buffalo preantral follicles	Maintained follicle viability and growth by providing surface interaction and increasing attachment of follicles.	
	Kossowska-Tomaszczuk [205]	2010	A three-dimensional culture system containing type I collagen	Immunodeficient mice	Allowed granulosa cell subpopulations isolated from mature follicles to survive and grow, and supported their proliferation into steroid-producing spherical structures.	
	Saori Itami [206]	2011	A three-dimensional collagen gel	Mouse preantral follicles	The follicle could maintain its three-dimensional shape, and increase its size in response to FSH stimulation.	
	R Abir [201]	1999	collagen gel	Monolayer follicles from human ovarian tissue	Reported an increase in the GC layer and oocyte diameter of human follicles.	
	Catherine M H Combelles [202]	2005	3D collagen gel matrix	Cumulus cells	Established for the first time an effective in vitro fertilization combined culture system of human denuded oocytes and cumulus cells.	
	L Vanhoutte [213]	2009	Collagen (type I) gel	Dermished foamed oocytes	The fertilization rate of 3D pre-cultured oocytes was significantly higher than that of conventional IVM oocytes.	
	Yanjun Yang [214]	2019	The collagen scaffold loaded with hUCMSCs	Cyclophosphamide-induced POF mouse model	Increased the levels of E2 and AMH, ovarian volume and the number of antral follicles.	
	Jing Su [154]	2016	The collagen scaffold with ADSCs	Tripterygium Glycosides -induced POF rat model	Increased long-term retention of ADSCs in the ovary and contributed to the restoration of ovarian function.	
	Lijun Ding [215]	2018	The collagen scaffold with umbilical cord mesenchymal stem cells	Infertile POF patients	Saved overall ovarian function and leaded to a successful clinical pregnancy.	
Hyaluronic acid	Nina Desai [219]	2012	A tyramine-based HA hydrogel	Mouse preantral follicles	Promoted the secretion of estradiol and increased the survival rate, GV rupture rate and MII formation rate of cultured follicles.	
	I R Brito [220]	2016	A novel hyaluronic acid hydrogel based on tyramine-substituted sodium hyaluronate dihydroxyphenyl bond	Goat preantral follicles	Failed to maintain survival and improve antral formation.	
	Parisa Jamalzaei [222]	2020	A HAA composed of HA and ALG	Mouse preantral follicles	Promoted the development of preantral follicles and oocyte maturation in mice and enhanced estrogen secretion.	
	L M G Paim [224]	2015	A vitrification solution with 1% hyaluronic acid	The cumulus oocyte complex	Improved the meiotic recovery rate and nuclear maturation rate of norvegicus oocytes.	
	Somayeh Tavana [225]	2016	The HABH	Ovariectomized rats	Prevented or reduced early ischemia-induced follicular loss, promoted follicular survival and angiogenesis.	
	Maryam Akhavan Taheri [226]	2016	HA hydrogel	Ovariectomized rats	Had no negative effect on estrus cycle recovery and ovarian preservation,and improved the outcome of autologous transplantation.	
	Or Friedman [227]	2012	HA—rich biogel	Immunodeficient mice	Improved ovarian graft survival.	
	Wenlin Jiao [228]	2022	A combination of UCMSCs and HA gel	4-Vinylcyclohexene diepoxide -induced POI mouse model	Improved follicular survival.	
	Eun-Young Shin [229]	2021	HA gel scaffolder	Cisplatin-induced POI mouse model	Restored the ovarian structure and function and improved the quality of oocyte and embryo as well as the regularity of estrus cycle.	
	Guangfeng Zhao [230]	2015	HA	Immunosuppressive drug-induced POI-like rat model	Prevented chemotherapy-induced ovarian damage.	
Fibrin	Seyedeh Zeynab Sadr [235]	2018	Fibrinalginate scaffold	Mouse preluminal follicles	Improved follicular development and survival, and produced mature oocytes.	
	Shi Ying Jin [240]	2010	A fibrinalginate hydrogel matrix	Mouse secondary follicles	Supported the growth of secondary follicles to the antral follicles stage and produced mature oocytes.	
	Ariella Shikanov [239, 241]	2011 2009	The FA-IPN	Mouse secondary follicles	Contributed to increased meiosis maturation rates of oocytes.	
	I R Brito [220]	2016	Fibrinalginate	Goat preluminal follicles	Restored oocyte meiosis and promoted oocyte maturation to produce parthenotes.	
	J Xu [242]	2011	A fibrin alginate matrix	Rhesus monkey secondary follicles	Supported the growth of secondary follicles to antral follicles stage, and promoted the maturation of oocytes to MII stage.	
	J Xu [243]	2013	Fibrinin-sodium alginate 3D capsule	Primate rhesus monkey primary follicles	Primate oocytes derived from primary follicles developed in vitro had the ability to restart meiosis for fertilization.	
	Alireza Rajabzadeh [245]	2020	A fibrin hydrogel scaffold supplemented with platelet lysates	Mouse preantral follicles	Improved the local vascularization of follicles, and the survival rate of follicles, and promoted the growth of follicles to the stage of antral follicles.	
	Valérie Luyckx [246]	2014	A fibrin matrix containing low concentrations of fibrinogen and thrombin	Mouse preantral follicles and ovarian cells	All follicles were found to be alive or only slightly damaged and to grow to the antral follicular stage.	
	M C Chiti [247]	2016	Fibrinogen and thrombin (F12.5/T1) substrates	SCID mice	Isolated secondary follicles survived and grew to the antral follicle stage.	
	Rachel M Smith [248]	2014	Fibrin hydrogel	Infertile mouse model	Restored ovarian endocrine function.	
	Fernanda Paulini [249]	2016	A fibrin matrix containing fibrinogen and thrombin	Nude mice	Isolated human follicles were viable after encapsulation in fibrin clots and short-term xenotransplantation.	
	Ariella Shikanov [244]	2011	Heparin modified fibrin	Infertile mouse model	Reduced ischemia and improved vascular remodeling.	
	Jiang-Man Gao[250]	2013	Fibrin hydrogels mixed	Adult female mice	Increased follicular survival and improved revascularization.	
	Chungmo Yang [251]	2021	Fibrin hydrogel containing NO-NPs	Ovariectomized mice	Improved the total number and quality of follicles, induced angiogenesis, and prevented ischemic injury.	
	Elham Shojafar [252]	2019	Platelette-rich fibrin biofolders	Ovariectomized mice	Reduced oxidative stress, promoted revascularization, and protected follicular cisterns from ischemia–reperfusion injury.	
	Maria Costanza Chiti [237]	2018	A novel fibrin matrix	Human ovarian follicles	Fibrin matrix composed of F50/T50 most closely resembled human ovarian cortex.	
	Valérie Luyckx [238]	2013	A artificial ovary composed of fibrinogen and thrombin	Human ovarian cells	Enabled the survival and proliferation of isolated human ovarian stromal cells.	
Alginate	Hudson H V Correia [259]	2020	A sodium alginate 3D culture system	Goat primordia follicles	Showed appropriate survival rate, high follicular activation rate and continued to grow throughout culture.	
	Samaneh Sadeghnia [261]	2016	A sodium alginate three-dimensional culture system	Sheep primordial/primary follicles	2% sodium alginate supported follicle growth better than 1% sodium alginate.	
	Min Xu [262]	2006	An alginate hydrogel matrix	Pseudopregnant female mice	Produced healthy and fertile progenies.	
	Jing Xu [263]	2010	Alginate	Rhesus monkey secondary follicles	Grew to the antral follicle stage, produced steroids and growth factors, and produced healthy oocytes within 40 days.	
	Min Xu [264]	2009	Alginate	Rhesus monkey secondary follicles	The follicles survived and continued to grow.	
	Alon Kedem [266]	2011	Macropores sodium alginate scaffold	Human ovarian cortex slices	There was an increase in developing follicle culture and a decrease in atretic follicles.	
	Monica M Laronda [267]	2014	Sodium alginate hydrogel	Human ovarian cortex containing primordial follicles	The ovarian cortex grew, survived, and supported follicular development for up to 6 weeks.	
	Christiani A Amorim [268]	2009	alginate matrix	Small human preantral follicles	Survived in vitro culture in alginate matrix for 7 days.	
	Antonella Mastrorocco[269]	2021	Alginate microspheres	Lamb cumulus oocyte complexes	Increased the nuclear maturation rate of preadolescent oocytes and reduced the incidence of chromosome abnormality.	
	Parisa Jamalzaei [270]	2020	ALG hydrogel	Mouse preantral follicles	Survival rate of 0.5%ALG cultured follicles was significantly higher than 0.75% and 1%ALG cultured follicles.	
	Cyrus Jalili [271]	2020	Sodium alginate	Mouse preantral follicles	0.5% alginate was the most favorable concentration.	
	Erin R West[258]	2007	Alginate gel	Mouse secondary oocytes	Reducing alginate matrix hardness could maintain intercellular tension homeostasis, promote cell process, create local paracrine environment and improve oocyte quality.	
	Julie Vanacker [273]	2014	Alginate saline gel	Immunodeficient mice	Promoted follicular development and vascularization.	
	Sivanandane Sittadjody [274]	2017	Sr++ cross-linked alginate	Ovariectorized rats	Achieved stable hormone secretion and improved the adverse effects of hormone deficiency.	
	Shani Felder [275]	2019	Macrofenate scaffold	Ovariectorized mice	Showed high serum hormone levels and the appearance of the vaginal area.	
Sythetic biomaterials	Jiwon Kim [276]	2016	A synthetic hydrogel, PEG-VS	Ovariectorized mice	It was found to wrap immature follicles successfully functioned as an artificial ovarian tissue in vivo for 60 days.	
	Uziel Mendez [277]	2018	A three-dimensional PEG-based culture system	Mice follicles	Improved the survival and maturation rates of small follicles.	
	Zhonghua Shi [278]	2021	A supramolecular hydrogel	Aged mice	Delayed ovarian aging in aged mice,stimulated ovaries to secrete estrogen and progesterone, and developed more antral follicles for reproduction.	
	Anu David [279]	2017	A TheraCyte device	Ovariectomized mice	Restored follicular development and ovarian endocrine function and reduced FSH levels.	

### Application of biomaterials in the treatment of ovarian aging

#### Extracellular vesicles

**Fig. 6:**
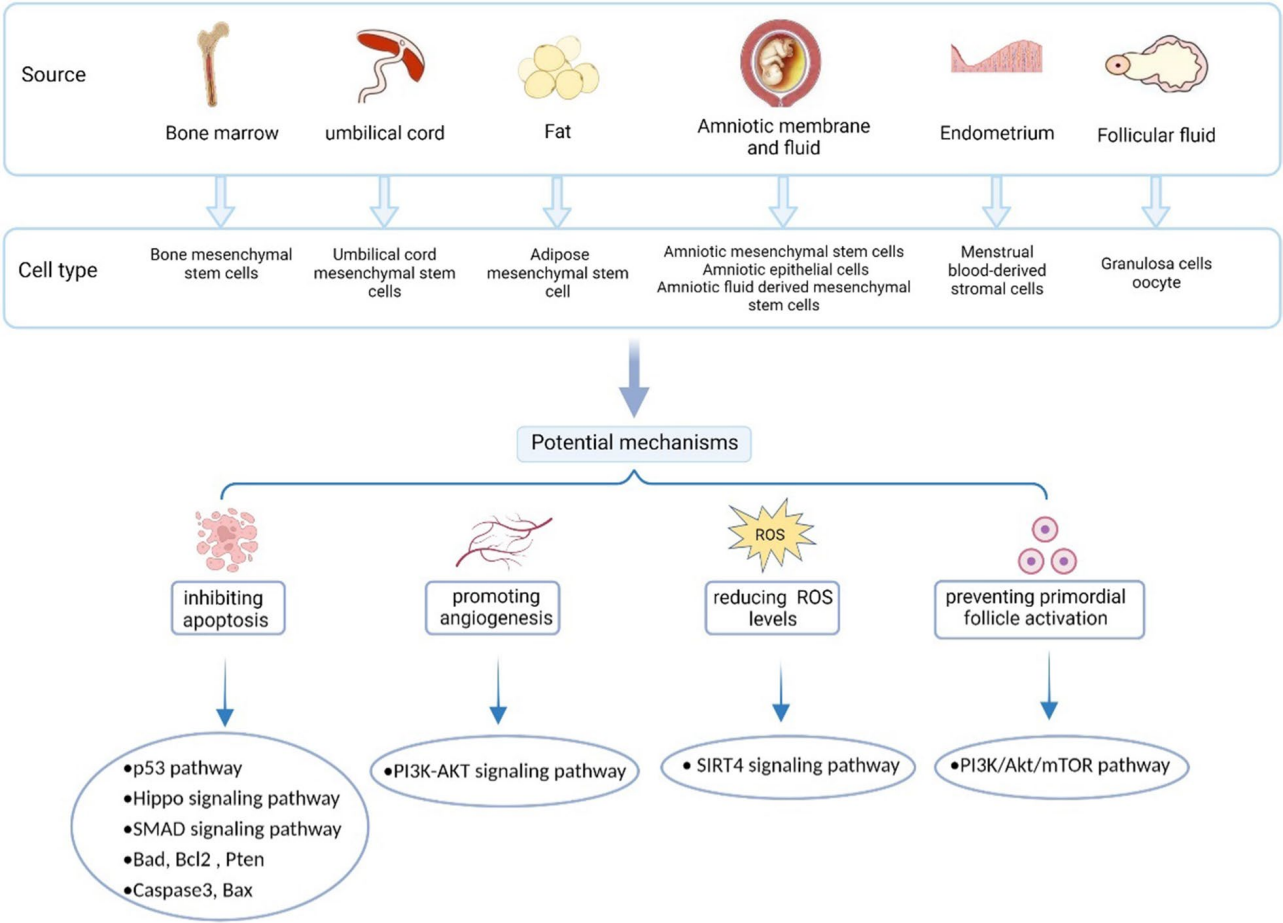
The therapeutic effects of stem cell-derived EVs in the treatment of ovarian aging

EVs, known as nano-sized, are a heterogeneous group of cell-derived membranous structures comprising exosomes (~ 50–150 nm) and microvesicles (~ 100–1000 nm), which carry bioactive material such as mRNAs, microRNAs (miRNAs), and protein in different body fluids and deliver their contents to recipient cells [142]. In recent years, EVs has developed into an effective nanocarrier for advanced drug delivery due to its multiple advantages [143]. EVs, as a natural vector produced by endogenous cells, have good biocompatibility with the immune system and low toxicity. In addition, EVs avoid phagocytosis by macrophages and penetrates blood vessels into the extracellular matrix. Furthermore, EVs can cross biological barriers to treat refractory diseases, such as the blood–brain barrier [144]. With the exploration of EVs, stem cell-derived EVs have attracted much scientific attention due to its broad prospects for treatment of various diseases. Regarding the function of EVs depends on their parent cells, accumulating studies have shown that stem cell-derived EVs can treat ovarian aging by transferring functional miRNAs and proteins. The following sections will mainly elucidate the therapeutic effects of stem cell-derived EVs in the treatment of ovarian aging (Fig. 6).

##### Bone marrow mesenchymal stem cell (BMSC)-derived exosomes

Bone marrow mesenchymal stem cells (BMSCs) are a cell subpopulation with multiple differentiation potential, and they constitute a popular research topic in the field of stem cell therapy [145]. Studies have shown that exosomes derived from BMSC (BMSC-exos) can also participate in tissue repair, and they are expected to replace stem cells as a new therapeutic tool for tissue repair [146]. It has been reported that miR-644-5p carried by BMSC-derived exosomes improved follicle injury and ovarian function by inhibiting the apoptosis of ovarian granulosa cells induced by cisplatin through targeting of the p53 pathway [145]. Their results suggested that inhibition of the apoptosis pathway in granulosa cells might occur via horizontal transfer of mRNAs by BMSC-exos. In another chemotherapy-induced POF rat model, the prominent role of the contents of BMSC-exos was studied. BMSC-exos exerted anti-apoptotic effects on tubular granulosa cells via the delivery of miR-144-5p, which regulated proliferative/anti-apoptotic pathways, leading to restoration of ovarian function [147].

##### Human umbilical cord mesenchymal stem cell (hUCMSC)-derived exosomes

The human umbilical cord is a promising source of MSCs, and hUCMSCs have a painless collection procedure and faster self-renewal properties. hUCMSC-derived exosomes (hUCMSC-exos) help to maintain tissue homeostasis and enable the recovery of critical cellular functions by initiating the process of repair and regeneration. hUCMSC-exos have also proven to be effective in recovering ovarian function and improving fertility in ovarian aging. Li et al. investigated the effect of hUCMSC-exos on POF induced by cyclophosphamide (CTX) in a mouse model [148]. The results showed that hUCMSC-exos could reduce cell apoptosis and enhance proliferation through the Hippo signaling pathway, leading to ovarian cells recovery and overall improvement of ovarian function. In a recent animal study, Liu et al. studied the effect of hUCMSC-exos on POI induced by chemotherapy in mice [149]. They demonstrated that hUCMSC-exos improved the fertility of POI mice by inhibiting the apoptosis of ovarian cells mediated by mRNAs and miRNAs transferred by the hUCMSC-exos. In another study, Yang et al. explored the proangiogenesis effect of hUCMSC-exos in a mouse model of POI [150]. They demonstrated that hUCMSC-exos transplantation could restore ovarian function by promoting angiogenesis through activation of the PI3K-AKT signaling pathway. hUCMSC-exos also exerted ovary protection via their anti-inflammatory effects. In a CTX-induced POI mouse model, injection of hUCMSC-exos reduced ROS levels by suppressing SIRT7 expression in the damaged ovary [151]. Furthermore, an in vitro study by Zhang et al. showed that hUCMSC-exos increased the proportion of Bcl-2/Bax and decreased the expression of the proapoptotic gene Caspase-3, therefore playing important roles in promoting resistance to cisplatin-induced granulosa cell apoptosis and restoring the synthesis and secretion of steroid hormones in granulosa cells [152]. In a similar study, hUCMSC-exos treatment ameliorated cisplatin-induced granulosa cell stress and apoptosis in vitro [153].

##### Human adipose mesenchymal stem cell (hADMSC)-derived exosomes

hADMSCs are derived from human adipose tissue and are superior biomaterials that can be suitable for allotransplantation and regenerative medicine [154]. Hung et al. established a mouse POI model by CTX administration to study the therapeutic effect of hADMSC-derived exosomes (hADMSC-exos) in chemotherapy-induced ovarian aging. The results showed that hADMSC-exos inhibited the expression of apoptosis-related genes in granulosa cells and improved ovarian function via regulation of the SMAD signaling pathway [155].

##### Human amniotic mesenchymal stem cell (hAMSC)-derived exosomes

hAMSCs are derived from the human amniotic membrane, are easy to obtain, are less invasive and are ethical, and they can be used in allotransplantation and regenerative medicine [156]. hAMSCs have been shown to be effective in recovering ovarian function in a mouse model of POF [156]. However, little is still known about the underlying molecular mechanism of hAMSC treatment in ovarian damage, and much remains to be further clarified. In another study, Ding et al. first reported that hAMSC derived exosomes (hAMSC-exos) reversed apoptosis in a chemotherapy-induced POF mouse model. This study indicated that miR-320 in hAMSC-exos reduced ROS levels via SIRT4 signaling to exert protective effects on ovarian function [157].

##### Human amniotic epithelial cell (hAEC)-derived exosomes

hAEC-based therapy mediates tissue regeneration in a variety of diseases, and increasing evidence has suggested that the therapeutic efficacy of hAECs mainly depends on paracrine action [158–160]. The effects of hAEC-derived exosomes (hAEC-exos) were investigated in POF induced by busulfan and cyclophosphamide in mice. hAEC-exos significantly improved ovarian function by ameliorating the granulosa cell apoptosis and preventing the ovarian vasculature damage. An in vitro study showed that hAEC-exos increased the expression of anti-apoptotic genes, such as Bad, Bcl2, and PTEN, and decreased the expression of pro-apoptosis genes, such as Caspase-3 and Bax, by transferring functional miRNAs, such as miR-1246 [14]. In addition, the results showed that hAEC-exos prevented primordial follicle activation in chemotherapy-treated mice through the PI3K/AKT/mTOR pathway.

##### Amniotic fluid-derived mesenchymal stem cell (AFMSC)-derived exosomes

AFMSCs are adult, fibroblast-like, self-regenerating pluripotent stem cells [161]. Accordingly, AFMSCs serve as a rich source of MSCs in terms of the number of potential donors and the simplicity of the harvesting procedure [162]. Based on their enormous differentiation capacity and immunomodulatory characteristics, the therapeutic potential of AFMSCs has been extensively explored in animal models of degenerative diseases. In a mouse model of POF induced by CTX, Xiao et al. showed that injection of AFMSC-derived exosomes (AFMSC-exos) protected mice from ovarian damage by reducing apoptosis of granulosa cells [163]. They revealed that AFMSC-exos contained two miRNAs, miR-146a and miR-10a, which inhibited apoptosis in damaged granulosa cells and prevented the atresia of ovarian follicles induced by CTX [163]. Thabet et al. showed that AFMSC-exos were able to repair CTX-induced POF in rats [164]. They found that AFMSC-exos containing miRNA-21 could inhibit the expression of target genes, such as PTEN and Caspase-3 in ovarian cells. These target genes are involved in the apoptosis and physiology of follicles [164].

##### Menstrual blood-derived stromal cell (MenSC)-derived exosomes

Mesenchymal stromal cells isolated from menstrual blood (MenSCs), exhibiting a potent proangiogenic and immunomodulatory capacity, have become an important source of stromal cells for cell therapy [165]. Their therapeutic effect is mediated by paracrine mediators released by their secretomes. Previous studies have shown that MenSCs play a paracrine role in ovarian therapy, such as promoting the number of follicles and ovarian angiogenesis and reducing the apoptosis of granulosa cells [142, 145]. Recently, Zhang et al. found that menstrual blood-derived stromal cell-derived exosomes (MenSC-exos) transplantation could effectively promote follicular development, restore fertility and improve live birth rates in a chemotherapy-induced POI rat model [166]. These protective effects might mainly be due to improvement of the ovarian extracellular matrix and the proliferation of granulosa cells.

##### Follicular fluid-derived exosomes

Follicular fluid has been recognized as a source of biochemical factors that can be predictive of oocyte quality, and it contains a variety of important secretory factors, such as proteins, amino acids, nucleotides, hormones and so on [167]. The microenvironment provided by follicular fluid plays an important role in follicular growth and maturation [168]. Follicular fluid exosomes are new molecules in follicular fluid, and they have been successfully isolated from human, bovine, and pig ovaries [169–171]. Juliano et al. proved that microvesicles isolated from follicular fluid could be taken up by surrounding granulosa cells [168]. Recently, Yuan et al. found that follicular fluid exosomes increased the proliferation and progesterone synthesis of porcine ovarian granulosa cells, in which the MAPK/ERK and WNT/B-CATENIN pathways were involved [172]. Another study investigated the role of the antioxidative properties of follicular fluid exosomes in bovine granulosa cells [173]. The results showed that follicular fluid exosomes had protective effects against heat stress by reducing the amount of ROS accumulation. In a similar study, to improve cumulus cell expansion and oocyte competence for fertilization, treatment with follicular fluid exosomes increased the resistance of oocytes to heat shock and improved the cleavage and blastocyst rates [174].

Together, EVs, especially exosomes, have attracted significant interest with regard to their use in the treatment of ovarian aging. EVs can be readily isolated from stem cells of various origins and carry biologically active molecules that can be transferred to target cells to exert their therapeutic effects. EVs prevent ovarian aging by promoting angiogenesis, modulating the immune system, suppressing cell apoptosis, and exerting many other beneficial effects. However, the functional mechanisms of EVs must be determined to take full advantage of EVs in ovarian aging therapy.

#### Extracellular matrix

Tissues and organs contain a mixture of cellular and noncellular components that form well-organized networks called ECM. The ECM not only provides a physical scaffold for cell embedding, but also regulates many cellular processes such as cell growth, migration, differentiation, survival, homeostasis and morphogenesis [175, 176]. The major constituents of ECMs are fibrous forming proteins, such as collagens, elastin, fibronectin (FN), laminins, glycoproteins, proteoglycans (PGs) and glycosaminoglycans (GAGs), which are highly acidic and hydrated molecules [177] and synthetic ECM is a promising material for tissue engineering [178]. The ovarian tissue engineering concept presents a 3D system for folliculogenesis resumption, supporting follicle survival and growth, providing a new strategy for the treatment of ovarian aging [73].

**Fig. 7:**
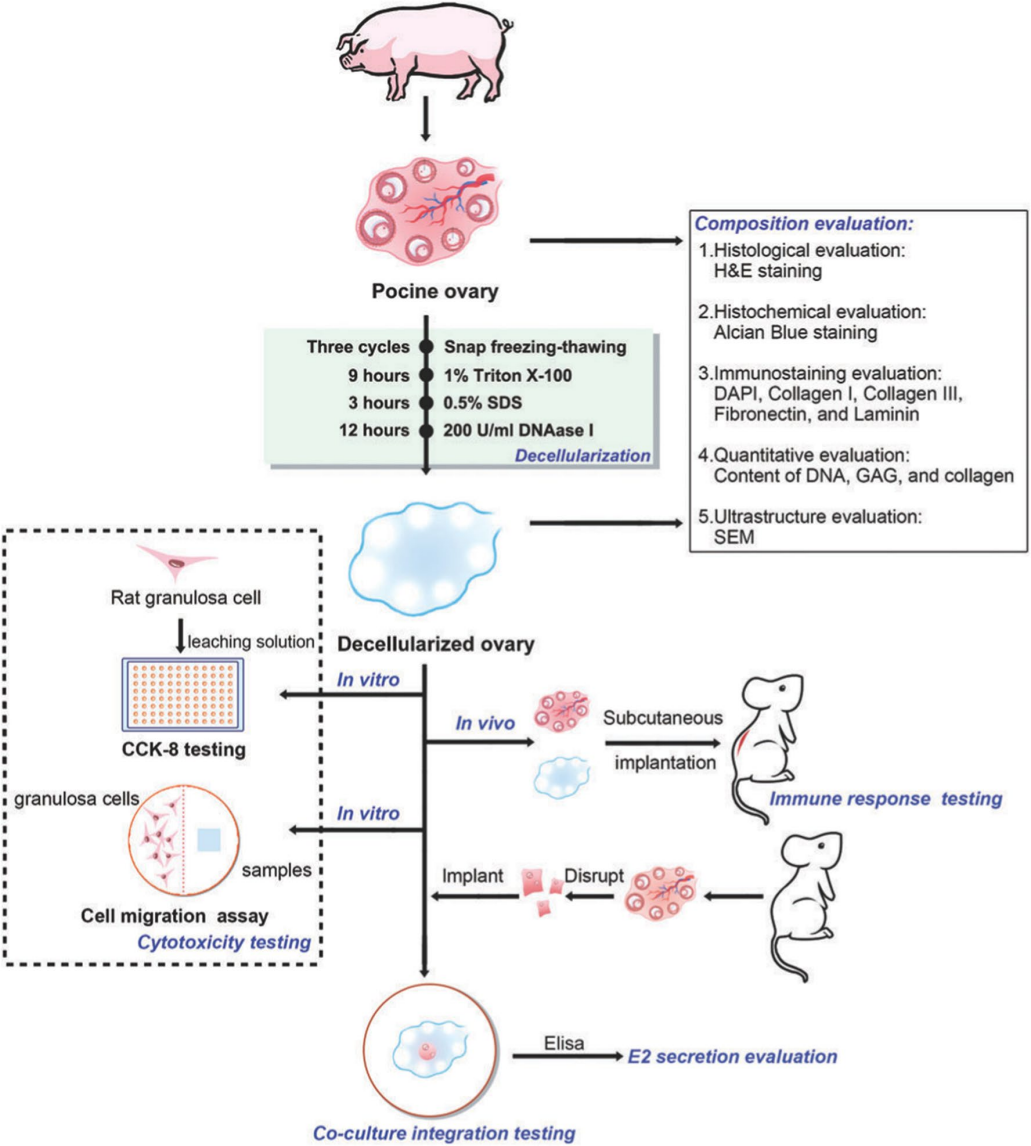
Flow diagram of study design. Color images available online at www.liebertpub.com/tec (the figure is reproduced from Liu et al. [187] with required copyright permission)

Detergents for the decellularization of whole organs or tissues are key factors in the preparation of acellular scaffolds with ECM structural integrity [179]. Originally, human and bovine ovaries were decellularized using sodium dodecyl sulfate (SDS) as an ionic detergent [180]. However, the long-term application of SDS can significantly change the ECM and has a strong, destructive effect on the ultrastructure of natural tissues, including the reduction of polysaccharides and cytokines, cytotoxicity, poor adhesion, and induced inflammation and thrombosis after transplantation [181–183]. Since then, the ovarian decellularization protocol has been improved. Studies have shown that the harmful effects of SDS are related to its concentration and exposure time [184, 185]. Pors et al. treated human ovarian tissue with 0.1% SDS as a cell detergent for 18–24 h, and they added DNA enzymes to decellularized human ovarian tissue and maintained ECM integrity [186]. Similarly, Liu et al. improved porcine ovarian decellularization strategies by adding Triton X-100 solution and shortening the SDS culture time, and further shortening the chemical treatment time by adding DNA enzyme digestion and freezing and thawing steps, and they developed a novel xenogenic ovarian regeneration decellularization protocol [187] as depicted in Fig. 7. Sistani et al. felt that the simplified procedure might better preserve the biochemical properties of the scaffold, so they applied three freezing/thawing cycles using a combined regimen of 1% Triton X-100 for 15 h and 0.5%SDS for 72 h without any enzyme treatment. The regiment could effectively decellularize human ovarian tissue and highly preserve ECM content and noncytotoxic properties [188]. Eivazkhania et al. demonstrated that sodium hydroxide (NaOH) could be used as a satisfactory decellularization agent for ovarian decellularization and regeneration of follicle-like structures [184]. Hassanpour et al. investigated a novel decellularization protocol based on sodium dodecyl sulfate (SLES) treatment, which avoided the disadvantages of SDS treatment, preserved the structure and composition of ovarian ECM, and promoted in vitro and in vivo biocompatibility and neovascularization of biological ovarian scaffolds [179].

Acellular tissue can improve follicular activity and growth by providing natural ECM components, growth factors and porous structures [189], making it an ideal scaffold for in vitro follicular culture. Nikniaz et al. cultured isolated mouse preantral follicles into an acellular ovarian scaffold and tested the survival rate of follicles for the first time, demonstrating that sodium alginate-containing acellular ovarian scaffolds could maintain follicular viability in vitro for 6 days [190]. Similarly, Alaee et al. cultured preantral follicles from mice in a decellularized rat ovarian scaffold for 12 days [191]. In the acellular ovarian scaffold, the preantral follicles transformed into antral follicles, and the mature follicles secreted E2 and progesterone (P4), and they could grow and develop normally and produce mature meiosis oocytes. Liu et al. implanted rat ovarian tissue into a porcine acellular scaffold, as depicted in Fig. 7. The acellular ovaries supported the adhesion, migration, and proliferation of immature female rat granulosa cells and showed estradiol secretion in vitro [187]. Pors et al. cultured human preantral follicles in vitro from acellular human ovarian tissue and demonstrated that the scaffold could support the survival of human follicles, while further research is needed to improve the recovery and survival of retransplanted follicles [186]. Kim et al. for the first time used ECM-derived hydrogels to perform 3D follicular culture in vitro and showed that this culture system could effectively improve the results of in vitro follicular culture, support follicular morphology and growth, and promote oocyte maturation [192].

Reconstruction of bioengineered ovaries could pave the way for possible in vitro reconstruction of ovarian tissue, which could in turn lead to overall improvements in reproductive technology and possibly future applications in organ transplantation to restore hormonal and reproductive function. Hassanpour et al. created artificial ovaries by implanting rat primary ovarian cells into an acellular scaffold of human ovarian tissue and implanted them into ovariectomized mice. Increased vaginal opening and estrogen levels after implantation confirmed the recovery of puberty [179]. Similarly, Laronda et al. showed that acellular bovine ovarian scaffolds supported the growth of isolated mouse follicles, produced estrogen and reconstructed menstrual cycles in ovariectomized mice [180]. Recently, Pennarossa et al. created a complete porcine ovarian 3D biological scaffold and refilled the ECM scaffold with female germline stem cells (FGSCs) to form a bioengineered ovary [193]. Notably, pregnancies have been reported following minimally invasive transplantation of previously cryopreserved ovarian tissue using human extracellular tissue matrix scaffolds assisted by robotic surgery [194].

#### Collagen

Collagen, which belongs to the fibrin family, is the most abundant extracellular matrix protein in the animal kingdom. It transmits loads in tissues and provides a highly biocompatible environment for cells [195]. This good biocompatibility, biodegradability, low inflammatory response, and low antigenicity make collagen a perfect biomaterial for regenerative medicine and tissue engineering [196].

There are 28 different members of the collagen family. Collagen type I, III and IV are the most abundant of the various collagen types in ovaries of vertebrates. The presence of normal collagen retains primordial follicle dormancy, follicles development, ovulation and steroidogenesis [197]. Therefore, collagen is a promising hydrogel for encapsulation of ovarian follicles. Joo et al. created collagen-rich, biomimetic 3D shells to culture rodent ovarian follicles [198]. They found that differences in cell survival, follicular growth and development, sex hormone production, and oocyte maturation were associated with changes in the density and elasticity of collagen hydrogel, suggesting that collagen hydrogel properties were important for follicular phenotype and function maintenance in 3D culture systems. In addition, follicles from several species have been successfully implanted into 3D collagen gel for culture. Torrance et al. developed a technique for isolating and growing intact mouse preantral follicles in a collagen gel matrix, and it allowed mouse follicles to separate and grow in vitro for at least 2 weeks [199]. Sharma et al. developed for the first time a 3D collagen gel culture system for the in vitro growth and survival of buffalo preantral follicles [200]. In addition, human [201, 202], pig [203] and bovine [204] follicles could be cultured in collagen gel, and it has been proven that the collagen gel culture system could provide maximal support for the growth of follicles, and maintaining their three-dimensional structure. Furthermore, a 3D matrix culture system consisting of type I collagen was constructed, which, together with leukemia inhibitors, allowed granulosa cell subpopulations isolated from mature follicles to survive and grow and supported their proliferation into steroid-producing spherical structures [205]. To study how the cell layer of the follicular membrane is formed, Itami et al. constructed a three-dimensional follicular culture system. Using this culture system, the follicles could maintain their three-dimensional shape by embedding in collagen gel, increasing their size in response to FSH stimulation, and replicating the formation of the cell layer of the follicle membrane when cultured with mesenchymal cells [206].

In vitro maturation (IVM) of human oocytes has the potential to provide some patients with the opportunity to receive fertility therapy; however, the conditions of human oocyte IVM remain to be improved [207]. Abir et al. embedded monolayer follicles from human ovarian tissue in collagen gel and cultured them for 24 h in vitro, establishing the first step of successful IVM of human small follicular oocytes. They reported for the first time an increase in the granulosa cell layer and oocyte diameter of human follicles isolated and cultured in collagen gel [201]. It is now clear that bidirectional communication between oocytes and their surrounding cumulus cells plays an important role in obtaining oocyte development capacity and subsequent embryogenesis [208–210]. Combelles et al. embedded cumulus cells into a 3D collagen gel matrix, adding a single oocyte to each gel, and they established an effective in vitro fertilization combined culture system of human denuded oocytes and cumulus cells [202]. In addition, it was found that the developmental potential of oocytes could be increased by temporarily inhibiting spontaneous meiosis maturation [211, 212]. Vanhoutte et al. precultured germinal foamed (GV) oocytes from the controlled ovarian overstimulation (COH) cycle in a collagen (type I) gel containing free cumulus cells and a specific phosphodiesterase 3 inhibitor (PDE3-I, inhibiting meiosis) for 24 h [213]. The results showed that the fertilization rate of 3D precultured oocytes was significantly higher than that of conventional IVM oocytes.

Indeed, the use of collagen as a scaffold for stem cell-based ovarian aging therapy is well documented. Yang et al. transplanted a collagen scaffold loaded with hUCMSCs into the ovaries of POF mice for the first time [214]. They demonstrated that the collagen scaffold increased the levels of E2 and AMH, the ovarian volume and the number of antral follicles. However, the mechanism of interaction between collagen scaffolds and stem cells remains unclear and requires further study. The collagen scaffold with hUCMSCs transplantation could represent an ideal and promising treatment for POF. In another study, Su et al. explored the transplantation of collagen scaffolds with adipose-derived stem cells (ADSCs) in a rat model of POF [154]. They observed that collagen scaffolds increased the long-term retention of ADSCs in the ovary and contributed to the restoration of ovarian function, including a regular estrus cycle, elevated E2 levels and improved fertility. These protective effects might be due to the growth factors secreted from ADSCs in the collagen scaffold, contributing to granulosa cell proliferation and angiogenesis within follicles. Although collagen scaffolds promoted the long-term retention of adipose stem cells in the ovary, the retention of these cells did not exceed 1 month and must be further optimized. Ding et al. showed that umbilical cord mesenchymal stem cells on collagen scaffolds (collagen/UC-MSCs) could activate primordial follicles in vitro by phosphorylation of FOXO3a and FOXO1 and activate follicles to grow to the preovulation stage in vivo [215]. In addition, they transplanted collagen/UC-MSCs into the ovaries of patients with POF, preserving overall ovarian function and a successful clinical pregnancy.

#### Hyaluronic acid

Hyaluronic acid (HA) is a biopolymer composed of disaccharide repeat units, including d-glucuronic acid molecules and *N*-acetylglucosamine molecules linked by B-(1–4) and B-(1–3) glycosides. It is present in all vertebrates and is an important component of the ECM in most mature tissues [216]. Hyaluronic acid has been widely used for its excellent physicochemical properties such as biodegradability, biocompatibility, non-toxicity, non-immunogenicity and as an excellent tool in biomedical applications such as osteoarthritis surgery, eye surgery, plastic surgery, tissue engineering and drug delivery [217].

In the ovary, HA is found not only in the cumulus matrix but also in the theca cells and follicular fluid, and they play a significant role in establishing a microenvironment conducive to the development of follicles [218]. Desai et al. described for the first time a novel tyramine-based HA hydrogel that supported the in vitro culture of mouse preantral follicles [219]. It was proven that oocytes from HA-encapsulated follicles could resume meiosis and produce mature MII oocytes. Brito et al. also used a novel HA hydrogel based on a tyramine-substituted sodium hyaluronate dihydroxyphenyl bond for the in vitro culture of caprine preantral follicles [220]. However, it was found that the follicles enclosed in HA failed to survive, possibly due to differences in experimental conditions (number of cultured follicles, culture medium and species). Compared with alginate (ALG), HA hydrogel lost the ability to increase follicular survival and the antral formation rate [220]. This outcome might be due to poor mechanical properties or a lack of pores in the HA microstructure, which are necessary for follicular nutrition and growth [221]. Jamalzaei et al. created a composite hydrogel (HAA) composed of HA and ALG to optimize the poor mechanical properties of HA and to form porous microstructures [222]. They found that the application of HAA hydrogel produced good results in follicular culture. Vitrification of embryos has been successful, while cryopreservation of oocytes has still failed to achieve the expected results [223]. Paim et al. used a vitrification solution with 1% hyaluronic acid to freeze the cumulus oocyte complex (COC) for 7 days and then heated and matured it in vitro for 30 h [224]. The results showed that adding 1% hyaluronic acid to vitrified frozen solution could improve the meiotic recovery rate and nuclear maturation rate of *Rattus norvegicus* oocytes in vitro.

Cryopreservation and transplantation of ovarian tissue is an effective method for the treatment of iatrogenic ovarian aging. Tavana et al. used a hyaluronic acid-based hydrogel (HABH) as a scaffold to improve ovarian tissue transplantation [225]. They found that ovarian encapsulation with HABH could prevent or reduce early ischemia-induced follicular loss and promote follicular survival and angiogenesis. However, the underlying mechanisms and clinical translational applications of HABH require further investigation. The same group used HA hydrogel as a scaffold to wrap vitrified ovarian tissue in autologous intramuscular transplantation, and they showed that it increased angiogenesis and reduced the follicular apoptosis rate in transplanted ovaries [226]. The results showed that the use of HA in combination with growth factors seemed to improve the outcome of autologous transplantation. Friedman et al. found that the coincubation of human ovarian grafts with HA-rich biogels in combination with vascular endothelial growth factor A (VEGF-A) and vitamin E resulted in improved ovarian graft survival [227].

Self-linked HA is a good cell scaffold to improve the transplantation of stem cells in the treatment of ovarian aging. Jiao et al. explored a combination of hUCMSCs and HA gel to rescue ovarian reserve and fecundity in a POI mouse model [228]. The HA gel not only increased the local retention of stem cells in the ovary, but also enhanced the paracrine function of hUCMSCs. The authors demonstrated that transplantation of hUCMSCs combined with HA gel could improve follicular survival by activating the PI3K-AKT pathway. Shin et al. transplanted embryonic stem cell-derived mesenchymal progenitor cells (ESC-MPCs) into cisplatin-induced POI mouse models by using HA gel scaffolds [229]. This method could effectively restore the ovarian structure and function of POI mice and improve the quality of oocytes and embryos, as well as the regularity of the estrus cycle. Interestingly, Zhao et al. demonstrated that HA supplementation prevented the occurrence of POI induced by treatment with the immunosuppressive agent tripterygium glycosides (TGs) [230]. This study indicated that HA promoted granulosa cell proliferation by upregulating PGRMC1 expression.

#### Fibrin

Fibrin (FIB), composed of fibrinogen and thrombin, is a natural scaffold formed after tissue injury, which can cause hemostasis and provide a useful initial matrix for cell adhesion, migration, proliferation and differentiation [231]. Fibrin has attracted the attention of tissue engineers because of its excellent biocompatibility, controllability and biodegradability as well as its ability to transfer cells and biomolecules. Fibrin is widely used in the development of cell-induced scaffolders [232], stem cell delivery [233] and induction of angiogenesis [234].

Fibrin by itself does not support follicular development in vitro because the encapsulated follicles secrete matrix-degrading proteolytic enzymes that cause follicle extrusion [235]. However, a combined culture system of fibrin alginate and fibrin thrombin can be successfully applied for in vitro follicular culture and artificial ovary construction [236–239]. Sadr et al. encapsulated mouse follicles in fibrin-alginate scaffolds and cultured them for 12 days. This culture system could improve follicular development and survival and produced mature oocytes [235]. Jin et al. isolated mouse secondary follicles and cultured them in a fibrin-alginate (FA) hydrogel matrix for 12 days [240]. The 3D culture system supported the growth of secondary follicles to the antral follicle stage and produced mature oocytes suitable for fertilization. Shikanov et al. developed a culture system based on the fibrin-sodium alginate interpenetration network (FA-IPN), which was subsequently used to grow mouse secondary follicles. This combination provided a dynamic mechanical environment that mimics the natural ovarian environment and contributed to increased meiosis maturation rates of oocytes [239, 241]. Brito used a culture system of FA to support the development of caprine preantral follicles, restore oocyte meiosis and promote oocyte maturation to produce parthenotes [220]. Notably, Xu et al. cultured isolated rhesus monkey secondary follicles in a fibrin alginate matrix for 40 days. The results showed that this culture system supported the growth of secondary follicles to the antral follicle stage in nonhuman primates and promoted the maturation of oocytes to the MII stage for the first time [242]. Subsequently, Xu et al. reported for the first time that fibrin-alginate 3D capsules could promote the development of primary follicles in primate rhesus monkeys and increase the production of follicle E2 and AMH in vitro [243]. In addition, they demonstrated that primate oocytes derived from primary follicles cultured in fibrin-alginate 3D capsules had the ability to restart meiosis for fertilization.

Cryo-thawed follicles have a better survival rate due to faster vascularization compared to the rate of ovarian tissue transplantation. The fibrin scaffold can control the release of growth factors and create a continuous path of cell infiltration between the host and graft [244], making it an ideal scaffold for follicular transplantation. Rajabzadeh et al. transplanted preantral follicles encapsulated in a fibrin hydrogel scaffold supplemented with platelet lysate [245]. The results showed that the culture system could significantly improve the local vascularization, survival rate, and growth of follicles. Luyckx et al. transplanted mouse preantral follicles and ovarian cells by wrapping them in a fibrin matrix containing low concentrations of fibrinogen and thrombin [246]. Almost all follicles were alive and grew to the antral follicular stage. The fibrin matrix also allowed for the proliferation of transplanted endothelial cells and capillary formation. Chiti et al. transplanted mouse primordial-primary and secondary follicles into severe combined immunodeficiency (SCID) mice by coating them with fibrinogen and thrombin (F12.5/T1) substrates. The results showed that isolated secondary follicles in the fibrin matrix could survive and grow to the antral follicle stage after short-term transplantation [247]. Smith et al. coated primordial follicles in fibrin hydrogel and transplanted them into an infertile mouse model [248]. The transplanted follicles could survive in infertile mice, develop into antral follicles and restore ovarian endocrine function. The surviving follicles were surrounded by the host interstitial tissue and produced luteum after ovulation. Paulini et al. xenografted human preantral follicles coated with a fibrin matrix containing fibrinogen and thrombin into the peritoneal sacs of nude mice [249]. The results showed that isolated human follicles were viable after encapsulation in fibrin clots.

Revascularization has always been an obstacle to the development of ovarian transplantation [244]. Shikanov et al. hypothesized that fibrin scaffolds provided a physical bridge between graft and host tissue and had the potential to enhance angiogenesis [244]. They transplanted vitrified/thawed ovarian tissue from mice coated with heparin binding peptide (HBP) and heparin-modified fibrin and loaded with vascular endothelial growth factor (VEGF) into infertile mouse models. The results showed that fibrin gel grafts could reduce ischemia and improve vascular remodeling after transplantation. The protocol also restored endocrine function and fertility in transplanted mice and allowed for natural conception. In addition, Gao et al. coated mouse ovarian tissues in fibrin hydrogels mixed with different concentrations of basic fibroblast growth factor (bFGF) and transplanted them under the skin of adult female mice for 1 week [250]. They demonstrated that bFGF and fibrin hydrogels could increase follicular survival and improve revascularization after ovarian transplantation. In addition, the high concentration of bFGF promoted the revascularization of transplanted ovarian tissue. Yang et al. transplanted mouse ovaries by wrapping them in fibrin hydrogels containing nitric oxide-releasing nanoparticles (NO-NPs) [251]. The results showed that the NO-NP/fibrin hydrogel improved the total number and quality of follicles after transplantation, induced angiogenesis, and prevented ischemic injury in the early stage of ovarian transplantation in mice. Shojafar et al. demonstrated that platelet-rich fibrin bioscaffolds indirectly reduced oxidative stress, promoted revascularization, and protected follicular cisterns from ischemia–reperfusion injury, thereby improving endocrine function and follicular formation in transplanted ovaries [252].

Fibrin-based scaffolders are also an alternative for the construction of artificial ovarian prototypes [253]. Chiti et al. compared the fiber thickness of four different fibrin formulations with the human ovarian cortex to optimize the composition of fibrin matrix and mimic the structure of human ovarian tissue, creating artificial ovaries that could grow human follicles [237]. Luyckx et al. reported that artificial ovaries formed by two optimal combinations of fibrinogen and thrombin (F12.5/T1 and F25/T4) enabled the survival and proliferation of isolated human ovarian stromal cells [238].

#### Alginate

Alginate is a group of non-branched polysaccharides composed of 1, 4-bound B-d-mannuronic acid (M) and A-l-guluronic acid (G) produced by brown algae and some bacteria [254]. Since alginate is non-toxic, rich in resources and easy to obtain, it is used as scaffold material for two-dimensional (2D) and 3D culture of mammalian cells [255]. In addition, alginate is able to form a soft hydrogel under physiological conditions, with pores large enough to allow nutrients and growth factors to pass through freely, while cells are trapped in the polymer network [256]. Their beneficial properties also include biocompatibility and biodegradability in human [257]. Based on the above advantages, alginate has become a very important biological material in pharmaceutical and biomedical fields.

Alginate hydrogels have been widely investigated in the culture of follicles from numerous animal species. The ultimate goal of an alginate system is to promote follicle development to obtain healthy oocytes that can be further matured and fertilized to produce embryos for fertility restoration [258]. Preantral follicles are the largest follicle population and represent an important source of potentially competent oocytes for further use in assisted reproductive technology [259]. There is evidence that the preantral follicle requires a hard tissue similar to the ovarian cortex to initiate in vitro development [260]. Alginate is an ideal scaffold for follicular culture in vitro and has been successfully applied in preantral follicle culture in many species. When Correia et al. coated goat primordial follicles in a sodium alginate 3D culture system, they showed an appropriate survival rate and high follicular activation rate [259]. Sadeghnia et al. evaluated sheep primordial/primary follicles in a sodium alginate three-dimensional culture system and found that the system supported the structural integrity of the follicles, with 2% sodium alginate supporting follicle growth better than 1% sodium alginate and increasing the diameter of the follicle [261]. The sodium alginate hydrogel matrix designed by Xu et al. promoted the follicular development of mature oocytes in vitro, and embryos extracted from cultured oocytes fertilized in vitro were transplanted into pseudopregnant female mice to produce healthy and fertile progeny [262]. In addition, studies have shown that, when coated with alginate and cultured, rhesus monkey secondary follicles could grow to the antral follicle stage and produce healthy oocytes for a long time [263, 264]. It is noteworthy that sodium alginate hydrogels can also be used for human ovary or follicle culture [265]. Kedem’s group tested the feasibility of culturing human ovarian cortex slices on macroporous sodium alginate scaffolds [266]. This study showed that there was an increase in the developing of follicle culture and a decrease in atretic follicles on sodium alginate scaffolds. Similarly, Laronda et al. coated human ovarian cortex-containing primordial follicles in sodium alginate hydrogel and found that the ovarian cortex grew, survived, and supported follicular development for up to 6 weeks in vitro [267]. Amorim et al. demonstrated that small human preantral follicles from frozen and thawed ovarian tissue could survive in vitro culture in alginate matrix for 7 days [268]. In addition, alginate culture systems can be used for IVM. Recently, Mastrorocco et al. developed a 3D IVM protocol for lamb COC encapsulated in the core of alginate microspheres [269]. This technique could increase the nuclear maturation rate of preadolescent oocytes and reduce the incidence of chromosome abnormalities, thus improving the in vitro performance of preadolescent lamb oocytes.

Jamalzaei et al. found that both the hardness and concentration of alginate ALG hydrogel affected follicle survival and found that the survival rate of 0.5%ALG cultured follicles was significantly higher than that of 0.75% and 1% ALG cultured follicles [270]. In addition, Jalili et al. compared the development of mouse preantral follicles in 3D media containing 0.25%, 0.5% and 1% sodium alginate [271]. They found that appropriate concentrations of sodium alginate hydrogels promoted follicular growth, maturation, and steroid hormones, with 0.5% alginate being the most favorable concentration. West’s group formed alginate brine gels of different hardnesses by changing the alginate solid concentration or by radiating or chemically oxidizing the polymer [258]. Secondary oocytes were coated with alginate gel with different hardnesses and growth, morphology and hormone secretion were observed. The results showed that reducing alginate matrix hardness could maintain intercellular tension homeostasis, promote cell processes, create a local paracrine environment and improve oocyte quality.

**Fig. 8:**
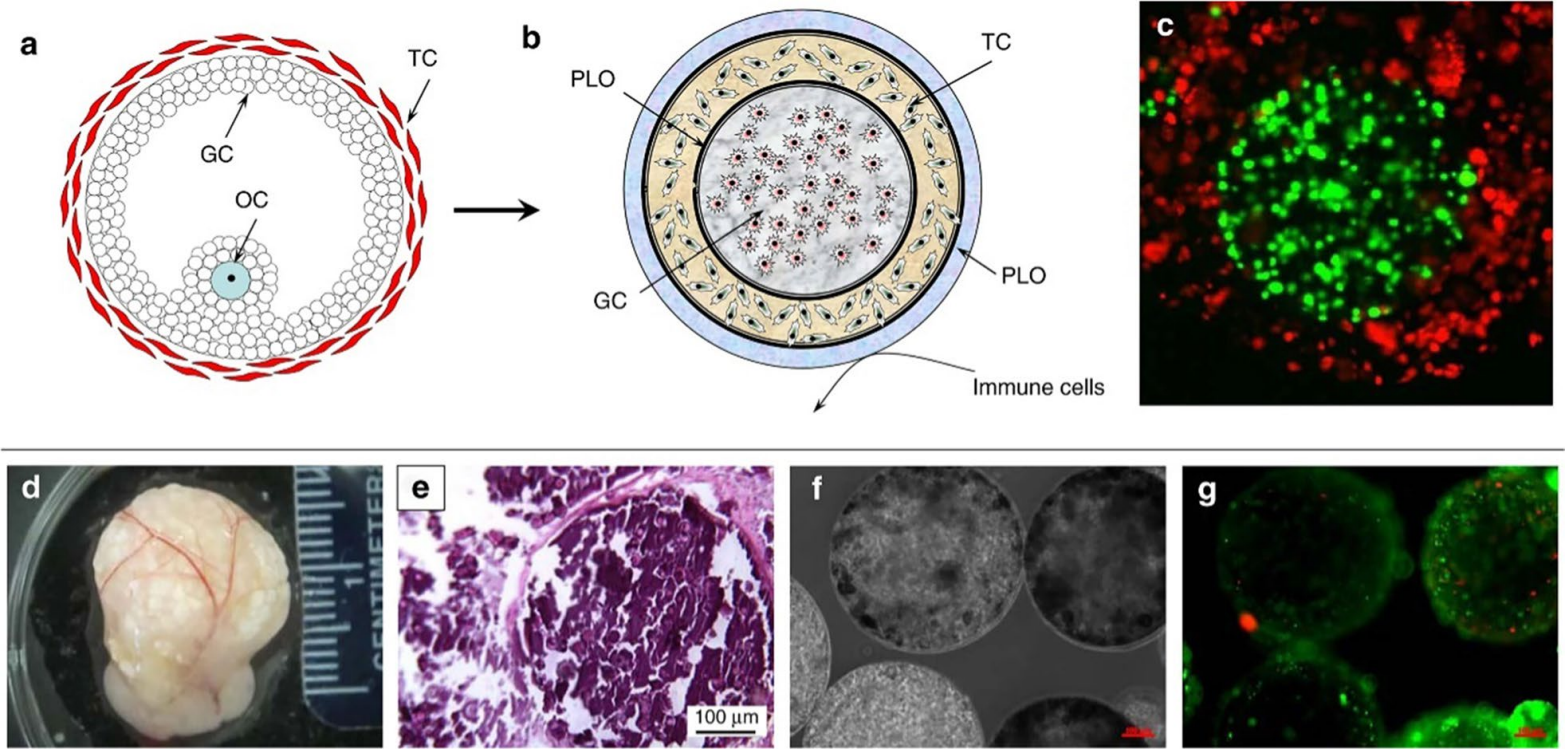
Schematic diagram of a native ovarian follicle (**a**) compared to the bioengineered ovarian construct (**b**). 3D-confocal images of bioengineered ovarian construct (**c**) demonstrating compartmentalization of different cells within the constructs as determined through the use of CellTracker green-labeled cells (granulosa) in the inner layer and CellTracker orange-labeled cells (theca) in the outer layer. Images of bioengineered ovarian construct retrieved 90 days after transplantation into ovariectomized rats including the presence of the vascularized omentum pouch enclosing the constructs following explantation (**d**). Explanted constructs showed minimal fibrous encapsulation as indicated by H&E staining (**e**). Phase-contrast images of the microcapsules after retrieval show that the constructs remain intact throughout the 90-day period tested in vivo (**f**). Live/dead imaging of the retrieved capsules (**g**), where green indicates live and red indicates dead cells, which shows that most cells in the constructs remained viable during the 90-day implantation period. Scale bars are 100 μm for **e**–**g** (the figure is reproduced from Sittadjody et al. [274] with required copyright permission)

Alginate not only can provide a supportive environment for implanted cells, allowing for full diffusion of nutrients and oxygen, but it also can act as a barrier between host and graft to prevent rejection caused by infiltration of host immune cells, making it suitable for ovarian transplantation [272]. For the first time, Vanacker et al. successfully constructed an artificial ovary with alginate saline gel encapsulated in mouse preantral follicles and transplanted it into immunodeficient mice [273]. The results showed that the artificial ovary promoted follicular development and vascularization. Sittadjody et al. constructed a 3D bioengineered ovary with Sr-cross-linked alginate and coated it with granulosa and theca cells by a cell encapsulation technique, as depicted in Fig. 8. After implantation in ovariectomized rats, the artificial ovary achieved stable hormone secretion for 90 days and improved the adverse effects of hormone deficiency, including osteoporosis, uterine hypertrophy and obesity [274]. In addition, Felder et al. constructed alginate scaffolds with affinity-bound bone morphogenetic protein-4 (BMP-4) to mimic the ovary microenvironment, and it supported the culture and growth of primordial follicles. The study showed the restoration of ovarian function in ovariectomized SCID mice after transplantation of this scaffold coated with porcine primordial follicles [275].

#### Synthetic biomaterials

Synthetic biomaterials can be tailored according to the physicochemical and mechanical properties of biological tissues, which have attracted great attention in the field of tissue engineering and regenerative medicine. At present, some studies also show that synthetic biomaterials play an important role in the recovery of ovarian function.

PEG is a commonly used biocompatible polymer that has been used to increase solubility, reduce accumulation, and prolong the blood half-life of various nanoparticles. Kim et al. engineered artificial ovarian tissue using a synthetic hydrogel, poly(ethylene glycol) vinyl sulfone (PEG-VS) as a supportive matrix [276]. The PEG-VS synthetic hydrogel was found to wrap immature follicles successfully and functioned as an artificial ovarian tissue in vivo for 60 days, demonstrating that PEG hydrogels provided a good microenvironment for follicles. Furthermore, Mendez et al. developed a three-dimensional PEG-based in vitro follicular culture system that could improve the survival and maturation rates of small follicles [277].

**Fig. 9:**
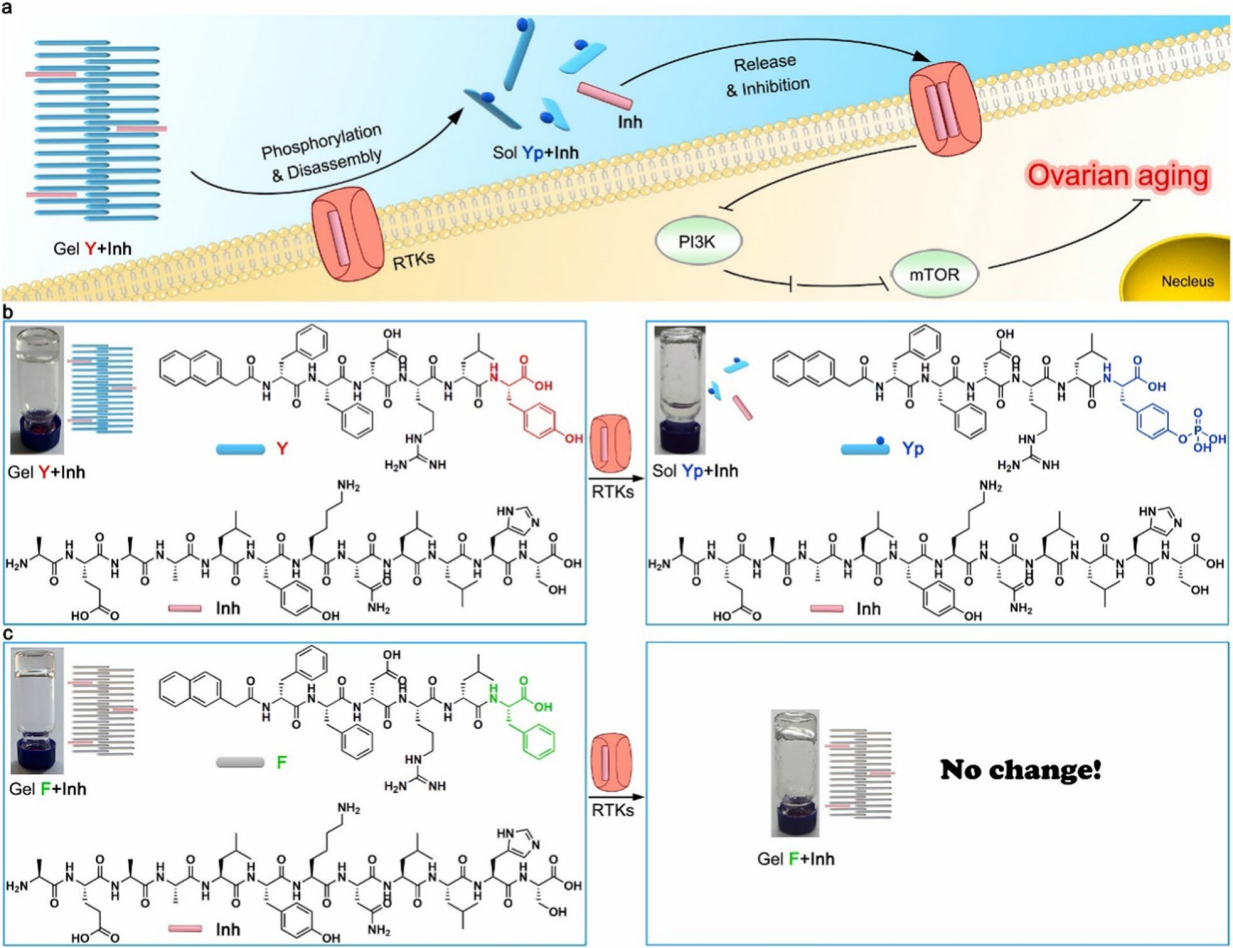
**a** Schematic illustration of RTK-instructed disassembly of hydrogel Gel Y + Inh for RTKs/PI3K signaling pathway inhibition. **b** RTK-instructed disassembly of Gel Y + Inh and the chemical structures of hydrogelator Y, its corresponding phosphate Yp, and a RTK inhibitor Inh. Photographs: Gel Y + Inh (left frame) and Gel Y + Inh incubated with SCFR (one type of RTKs) at 37 °C for 3 h (right frame). **c** Illustration of RTK-insusceptible hydrolgel Gel F + Inh and the chemical structures of hydrogelator F and Inh. Photographs: Gel F + Inh (left frame) and Gel F + Inh incubated with SCFR at 37 °C for 3 h (right frame) (the figure is reproduced from Shi et al. [278] with required copyright permission)

Supramolecular hydrogels, as a new type of soft biomaterial, have attracted extensive exploration because of their good biocompatibility and biodegradability. As shown in Fig. 9, Shi et al. designed a supramolecular hydrogel (Nap-Phe-Phe-Asp-Arg-Leu-Tyr-OH, Y)-coated receptor tyrosine kinase (RTK) inhibitor, called Gel Y + Inh (inhibitor of RTK), and developed an RTK responsive hydrogel to release Inh [278]. The results showed that the moderate release of Inh effectively delayed ovarian aging in aged mice by downregulation mTOR activity.

TheraCyte is an FDA-approved device made of a polytetrafluoroethylene (PTFE) membrane, which is impermeable to cells but allows for diffusion of soluble molecules through its 0.4-µm pores. David et al. transplanted a TheraCyte device coated with ovarian tissue into ovariectomized mice [279]. The TheraCyte device effectively isolated the graft from immune recognition and avoided the occurrence of immune rejection. The ovarian grafts encapsulated in TheraCyte devices could restore follicular development and ovarian endocrine function in ovariectomized mice.

Alhough synthetic biomaterials have been used in the treatment of ovarian aging, the low degradation rate, high hydrophobicity and low electrical conductivity of some synthetic polymer biomaterials remain major challenges in clinical application [280]. Conversely, the use of polymer biomaterials is often challenged by concerns about the lack of biological activity and foreign body reactions.

### Biomaterials applied to ovarian aging-related diseases

**Fig. 10:**
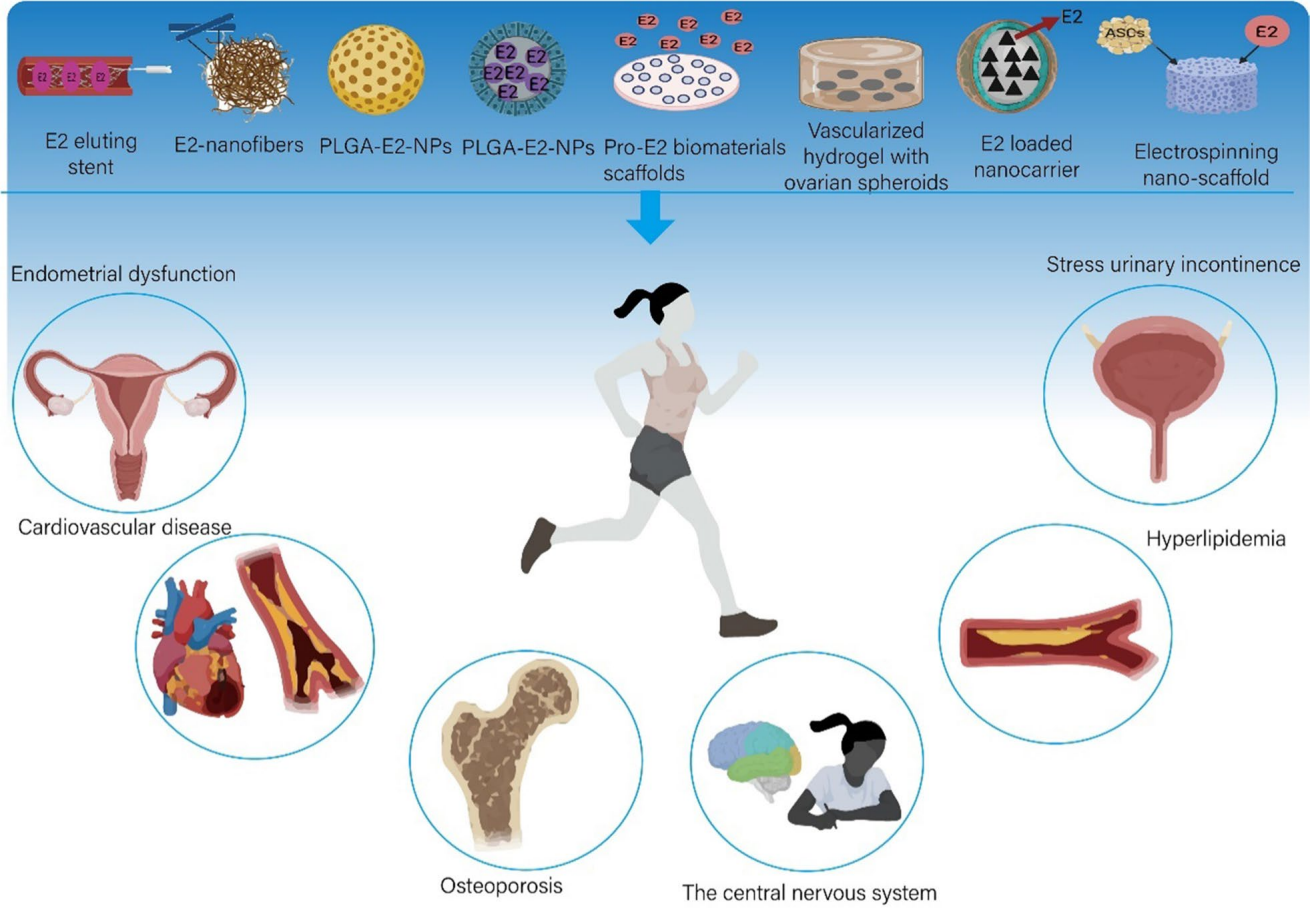
Biomaterials applied to ovarian aging related diseases

Ovarian aging can lead to endometrial dysfunction, cardiovascular disease, osteoporosis, central nervous system-related disease, hyperlipidemia, and stress urinary incontinence, seriously decreasing the quality of life of aged women. Based on the advantages and significant effects of biomaterials in the field of antiaging, different biomaterials have good effects on the diseases associated with ovarian aging. Next, we summarize the current application of biomaterials for the treatment of diseases related to ovarian aging (Fig. 10).

#### Endometrial dysfunction

Steroid hormones are the basis of normal endometrial function, and the initiation and regulation of endometrial shedding and repair are tightly controlled by these ovarian steroids [281]. When ovarian function is defective, insufficient secretion of steroid hormones will lead to endometrial dysfunction, such as a thin endometrium and infertility [282]. Yoon et al. used a rat ovariectomy model to harvest autologous ovarian cells to construct ovarian spherical vascularized hydrogels (VHOS) for hormone autocrination, and they implanted them into the hind limbs of ovariectomized rats [283]. Such VHOS could significantly promote the recovery of endocrine function and release of hormones, thus achieving complete regeneration of the endometrium.

#### Cardiovascular disease

Cardiovascular disease is the leading cause of death among women globally, and cardiovascular risk increases substantially following menopause. Premature menopause is associated with cardiovascular disease, with higher cardiovascular risks observed at progressively earlier menopausal ages [284]. Studies have shown that ovarian hormones, mainly estrogen, may play key roles in reducing the risk of heart disease [285]. Han et al. implanted 17β-E2 eluting stents into the abdominal aortae of rabbits fed a high-fat diet. They observed that the implantation of 17β-E2-coated stents inhibited ERK activation and reduced the formation of new intima after angiotensin conversion, thereby preventing restenosis [286]. Similarly, New et al. assessed the effect of 17β-E2-eluting stents on neovascularization in a pig model and showed that 17β-E2-eluting stents had potential benefits in the prevention and treatment of in-stent restenosis [287].

#### Osteoporosis (OP)

**Fig. 11:**
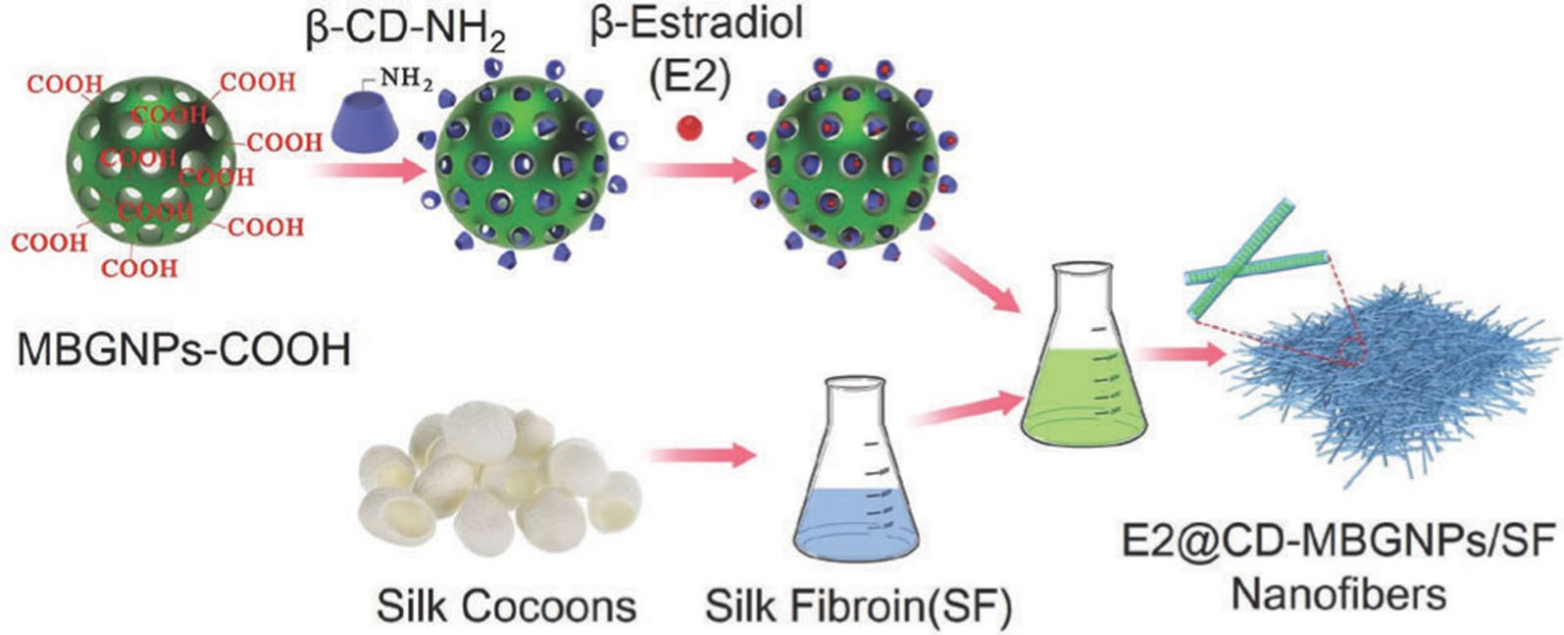
The schematic diagram of the design and preparation of beta-cyclodextrin modified mesoporous bioactive glass nanoparticles/silk fibroin hybrid nanofibers. (the figure is reproduced from Wang et al. [291]with required copyright permission)

Osteoporosis is a common disease in postmenopausal women characterized by reduced bone mass, deterioration of microstructures, and brittle fractures, affecting nearly one-third of women after the age of 50 [288]. Postmenopausal osteoporosis is largely the result of quantitative and qualitative bone changes caused by estrogen deficiency. Guo et al. constructed a drug delivery system based on PLGA nanoparticles (NPs), containing 17β-E2 and ferric oxide (Fe3O4) and modified it with alendronate sodium to achieve bone targeting and magnetic remote drug delivery [289]. In an osteoporosis model in ovariectomized (OVX) rats, the NPs showed a high encapsulation ability of E2 and were enriched in bone tissue. The three-month study showed that the NPs improved OVX-induced bone loss, increased bone strength and induced new bone formation with fewer adverse effects on other tissues. In another study, Takeuchi et al. explored an alternative administration route in a rat model of ovariectomized osteoporosis [290]. They prepared a transdermal delivery system of E2-loaded PLGA NPs for the treatment of osteoporosis. The results showed that E2-loaded PLGA NPs could effectively restore the bone mineral density of cancellous bone and prolong the interval between E2 administrations. In addition, Wang et al. developed a localized E2 delivery system incorporating β-cyclodextrin (CD-MBGNPs) and silk fibroin to sustain the constant release of E2 [291], as Fig. 11. The system could be utilized as a bone void filler for localized E2 delivery and bone regeneration in osteoporotic patients.

#### The central nervous system

**Fig. 12:**
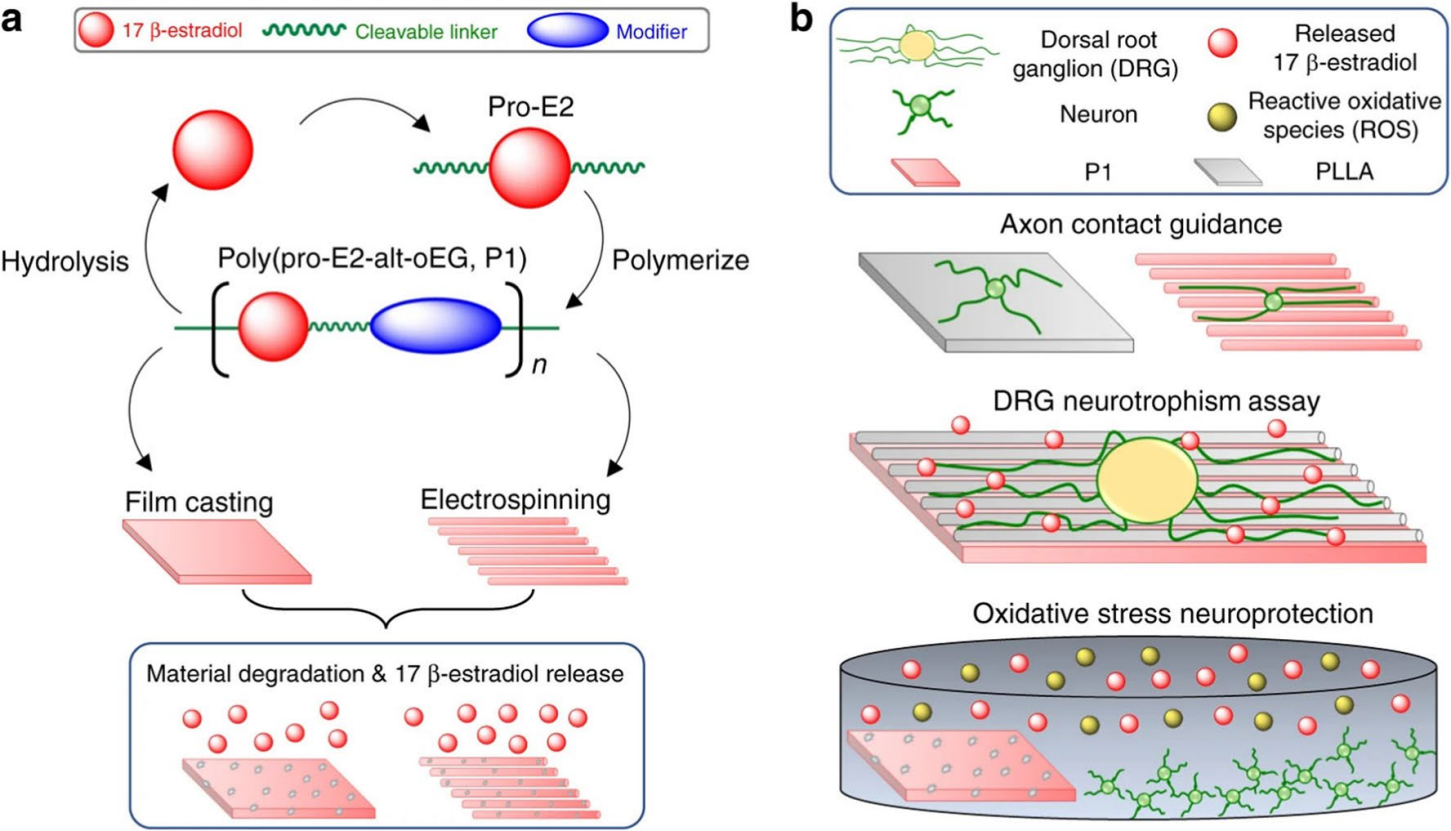
**a** Step-growth polymerization is used to synthesize P1 from pro-E2 and PEG dithiol monomers to create a copolymer that can be processed into thin films and electrospun fibers that deliver E2 as they degrade via hydrolysis. **b** Three different neuron culture models are used to demonstrate that electrospun P1 fibers provide contact guidance for extending neurites, and P1 films are neurotrophic and neuroprotective against oxidative stress (the figure is reproduced from D'Amato et al. [298] with required copyright permission)

Decreased estrogen levels before, during and after menopause can affect memory and cognitive function [292]. Alzheimer's disease (AD) is the most common neurodegenerative disorder, and female sex is a key risk factor for AD, especially in postmenopausal women [293]. In addition, there has been considerable evidence that estrogen has important neuroregulatory and neuroprotective effects [294]. Therefore, estrogen could be a potential treatment for ameliorating postmenopausal degenerative diseases of the central nervous system [295]. One study by Kreuter showed that PLGA micro/nanocarriers could enhance the therapeutic activity of E2 in the central nervous system (CNS) [296]. Prakapenka et al. subcutaneously injected E2-loaded PLGA NPs into rats undergoing menopause induced by OVX surgery and evaluated spatial learning and memory [297]. They demonstrated that delivery of E2 from PLGA NPs could enhance the beneficial cognitive effects of E2 relative to free E2 or non-hormone loaded nanoparticle controls. As shown in Fig. 12, D'Amato et al. designed a biomaterial scaffold composed of electrospun fibers and films completely composed of poly (pro-E2), which released E2 locally during in vitro for a long period of 1–10 years [298]. These scaffolds demonstrated the ability to promote and guide neurite extension and protect neurons from oxidative stress damage.

#### Hyperlipidemia

Menopause is associated with potentially adverse changes in serum lipids and lipoproteins, likely partially explaining the increase in cardiovascular disease (CVD) risk following menopause [299]. Estrogen deficiency due to ovarian function loss during menopause is a major cause of abnormal lipid metabolism. Improving estrogen levels is a promising approach for treating menopausal hyperlipidemia. Mittal et al. designed 17β-E2 capsule NPs to evaluate their efficacy in treating postmenopausal dyslipidemia in a rat model of hyperlipidemia induced by ovariectomy with a high-fat diet [300]. The results showed that these NPs improved the bioavailability of E2 and its effect on hyperlipidemia and alleviated the adverse effects of traditional hormone replacement therapy.

#### Stress urinary incontinence

Stress urinary incontinence (SUI) refers to the involuntary loss of urine in the absence of any prior sensation or need to empty; it occurs during physical activity and affects more than 50% of postmenopausal women [301]. The increased incidence of SUI in postmenopausal women is thought to be associated with decreased levels of 17β-E2 [301]. ADSCs are considered a promising method for the treatment of intrinsic sphincter deficiency due to urethral sphincter weakness and subsequent sphincter reconstruction [302, 303]. 17β-E2 has been shown to regulate the multidifferentiation ability of stem cells in bone, muscle, cartilage, and adipose tissue [304–306]. Based on this information, Feng's group developed a poly(l-lactide)/poly(e-caprolactone) electrospun nanoscaffold to combine ADSCs and E2 [307]. They found that the biocompatible cell/nanoscaffold with E2 could enhance the proliferation and myogenic differentiation of ADSCs and might be a feasible new option for SUI treatment.

## Practical challenges with biomaterials for the clinical evaluation and treatment of ovarian aging

It is evident that a number of preclinical studies have described the potential clinical value of biomaterials in treating ovarian aging. However, despite the theoretical benefits of biomaterials in treating ovarian aging and improving ovarian function, practical challenges remain in translating them into clinical practice.

First, safety is a major concern. As an organ to exercise fertility, the ovary not only affects women's own health but also plays an important role in the safety of offspring. Therefore, the materials used in the evaluation and treatment of ovarian aging must ensure their safety. Compared to natural biomaterials, it is challenging to obtain regulatory approval for synthetic biomaterials, and they might need to demonstrate additional safety and efficacy. The different physicochemical properties of NPs can also have adverse effects on humans, animal cells and invertebrate models, ultimately leading to toxicological consequences [308, 309]. For example, Sirotkin et al. compared the effects of different morphologies of copper nanoparticles (CuNPs) (spherical, triangular, and hexagonal) on the function of porcine ovarian granulosa cells [310]. The results showed that the cell viability of granulosa cells decreased after treatment with hexagonal CuNPs but increased after treatment with other CuNPs. Stelzer et al. demonstrated by coincubating rat ovarian granulosa cells that AuNPs could enter mammalian ovarian granulosa cells and affect steroid production. In addition, the ovaries contain a rich and highly porous network of blood vessels [311] so that biomaterials can easily accumulate in the ovaries through blood circulation [312]. Through in vivo experiments, Schadlich et al. detected the accumulation of nanoparticles, nanocapsules and nanolipid emulsions at specific locations in mouse ovaries [313]. Moreover, evidence accumulated from in vivo and in vitro models has suggested that the accumulation of nanoparticles in the ovary could an impair ovarian function by affecting sex hormone synthesis [314, 315], follicular development [31, 316] and oocyte quality [317, 318]. Therefore, the ovarian toxicity of NPs should be considered when selecting materials for the diagnosis and treatment of ovarian aging and a better examination of the ovarian toxicity of NPs will help to improve NPs regulation and design safer NPs.

Second, the effectiveness of biomaterials in the evaluation and treatment of ovarian aging must be clarified, and there is a lack of effective preclinical models to accurately predict the efficacy outcomes of these biomaterials. The effect of biomaterials on improving ovarian function is mostly based on the results of animal experiments. However, animal models differ from humans under study in biology, immunology and genetics, leading to the failure of biomaterials to successfully transition from animal to human therapy. For example, mouse follicles require only 350 μm in diameter to mature, while human follicles must be at least 5 mm. The alginate 3D culture system allows for follicle growth and maintenance in mice, but its nondegradability limits follicle growth in humans [277]. Moreover, the properties of biomaterials themselves also affect their effectiveness. Hyaluronic acid hydrogels cannot provide nutritional support for follicular growth due to their poor mechanical properties or the lack of pores in their microstructure [221]. Fibrin itself is easily degraded by proteolytic enzymes secreted by enveloping follicles and therefore cannot support follicular development in vitro [235]. Furthermore, the long-term effectiveness of the biomaterials in the treatment of ovarian aging must be confirmed. Nikniaz et al. cultured isolated mouse antral follicles in vitro for only 7 days [190], which was too short to evaluate follicular development. Pors et al. reseeded human antral follicles on decellularized scaffolds and grafted them subcutaneously into immunodeficient mice for 3 weeks [186]. However, the three-week transplant period was not sufficient for prolonged follicular development in humans. In addition, immune rejection affects the effectiveness of biomaterials. Biomaterials are usually classified as materials placed in the body, not only as materials in contact with the outside of the body [319]. As exogenous substances, biomaterials could activate the immune system to attack them and cause immune rejection. Hassanpou et al. established a human decellularized ovarian scaffold and found that residual cellular substances and cytotoxic detergents in the ECM of the recellularized scaffold might promote some immune cell infiltration within the graft [179]. Therefore, the efficacy of some materials in the treatment of ovarian aging must be improved.

Other potential challenges of biomaterials for the clinical evaluation and treatment of ovarian aging cannot be ignored. For example, moving biomaterials from the laboratory and into clinical use requires demonstrating biocompatibility with human tissue, which in turn requires additional years of preclinical studies. In addition, the therapeutic mechanisms of many biomaterials in ovarian aging are unclear, which could limit their clinical translation. Another challenge to clinical translation is the FDA approval process, because nanomedicines are the most heavily regulated consumer products throughout the premarket and postmarket phases.

Therefore, the clinical transformation of biological materials used for the evaluation and treatment of ovarian aging requires a long period of scientific research to achieve. With the progress of future science and technology, it could be possible to innovate and transform safe and novel biological materials to meet clinical needs.

## Conclusion and perspective

Timely diagnosis and treatment of ovarian aging and its related diseases have become urgent needs to improve the quality of life of women. This review highlights that the use of biomaterials could provide new directions for the diagnosis and treatment of ovarian aging. We have summarized innovative strategies for the evaluation and treatment of ovarian aging with different biomaterials. First, biological materials were constructed to detect hormone levels and were used for ultrasonic molecular imaging technology to achieve dynamic monitoring of ovarian function with high sensitivity and specificity. Furthermore, scientists have utilized the excellent properties of biomaterials to treat ovarian aging, such as improving the maturation and fertilization rates of oocytes, enhancing the treatment efficacy of stem cells, and developing artificial ovaries. Finally, different biomaterials used for the delivery of estrogen have good effects on the diseases associated with ovarian aging. All of these different options represent promising, albeit still experimental, strategies for improving ovarian aging. However, problems such as safety and effectiveness issues, lack of effective preclinical models, and unclear therapeutic mechanisms bring challenges to the clinical transformation of biomaterials. With advances in the regulatory mechanisms of biomaterials and enhanced safety assessments, biomaterials in the management of ovarian aging could be part of an exciting new strategy.

## Data Availability

Not applicable.

## Abbreviations

HRT: Hormone replacement therapy
ESCs: Embryonic stem cells
MSCs: Mesenchymal stem cells
iPSCs: Induced pluripotent stem cells
AMD: Age-related macular degeneration
PLGA: Poly lactic-co-glycolic acid
AuNPs: Gold nanoparticles
POF: Premature ovarian failure
DNA: Deoxyribonucleic acid
PM: Particulate matter
PAHs: Polycyclic aromatic hydrocarbons
POA: Premature ovarian aging
HPO: Hypothalamic pituitary ovarian
AITD: Autoimmune thyroid disease
GnRH: Gonadotropin-releasing hormone
IL-1α: Interleukin-1 alpha
IFN-γ: Interferon gamma
TNF-α: Tumor necrosis factor alpha
POI: Premature ovarian insufficiency
ROS: Reactive oxygen species
DDR: DNA damage response
MCM8: Minichromosome maintenance deficient 8
MCM9: Minichromosome maintenance proteins 9
MEIOB: Meiosis specific with OB domains
MND1: Meiotic nuclear divisions 1
PSMC3IP: Proteasome 26S ATPase subunit 3-interacting protein
HFM1: ATP-dependent DNA helicase homolog
MSH5: MutS protein homolog 5
IVF: In vitro fertilization
AMH: Anti-Müllerian hormone
AFC: Antral follicle count
TERT: Telomerase reverse transcriptase
RNA: Ribonucleic acid
H3: Histone H3
DOR: Diminished ovarian reserve
PLA: Polylactic acid
PGA: Polyglycolic acid
PCL: Polycaprolactone
PEG: Polyethylene glycol
EVs: Extracellular vesicles
NLP: Non-conjugated luminescent polymers
FDA: Food and Drug Administration
MRMD2.0: A Python tool for machine learning
TGF-β: Transforming growth factor β
ELISA: Enzyme linked immunosorbent assay
NBAMH: AMH-targeted nanobubbles
NBs: Nanobubbles
E1: Estrone
E2: Estradiol
E3: Estriol
UV: Ultraviolet
17β-E2: 17β-Estradiol
FSH: Follicle stimulating hormone
SINW-FET: Silicon nanowire field effect transistor
H2N–Fe-MIL-101 MOFs: Amine-functionalized iron-containing metal–organic framework
NicF: Porous nickel foam
Ab-FSH: FSH antibody
NiCo2O4/rGO: A novel nanomaterial
ITO: Indium tin oxide
mRNA: Messenger RNA
miRNA: Micro RNA
BMSCs: Bone marrow mesenchymal stem cells
BMSC-exos: Exosomes derived from bone marrow mesenchymal stem cell
p53: Tumor protein p53
MSC: Mesenchymal stem cell
hUCMSCs: Human umbilical cord mesenchymal stem cells
hUCMSC-exos: Human umbilical cord mesenchymal stem cell derived exosomes
CTX: Cyclophosphamide
PI3K: Phosphatidylinositol 3-kinase
AKT: Protein kinase B
SIRT7: Sirtuin-7
Bcl2: B-cell lymphoma-2
Bax: Apoptosis regulator BAX
Caspase: Cysteinyl aspartate specific proteinase
hADMSCs: Human adipose mesenchymal stem cells
hADMSC-exos: Human adipose mesenchymal stem cell derived exosomes
SMAD: Recombinant mothers against decapentaplegic homolog
hAMSCs: Human amniotic mesenchymal stem cells
hAMSC-exos: Human amniotic mesenchymal stem cell derived exosomes
SIRT4: Sirtuin-4
hAECs: Human amniotic epithelial cells
hAEC-exos: Human amniotic epithelial cell derived exosomes
Bad: Bcl-xL/Bcl-2 associated death promoter
PTEN: Gene of phosphate and tension homology deleted on chromosome ten
mTOR: Molecular target of rapamycin
AFMSCs: Amniotic fluid-derived mesenchymal stem cells
AFMSC-exos: Amniotic fluid-derived mesenchymal stem cell derived exosomes
MenSCs: Menstrual blood-derived stromal cells
MenSC-exos: Menstrual blood-derived stromal cell-derived exosomes
MAPK: Mitogen-activated protein kinase
ERK: Extracellular regulated protein kinases
WNT/B-CATENIN: Canonical Wnt/β-catenin pathway
ECM: Extracellular matrix
FN: Fibronectin
PGs: Proteoglycans
GAGs: Glycosaminoglycans
SDS: Sodium dodecyl sulfate
NAOH: Sodium hydroxide
SLES: Sodium dodecyl sulfate
P4: Progesterone
FGSCs: Female germ line stem cells
3D: Three-dimensional
IVM: In vitro maturation
GC: Granulosa cell
GV: Germinal foamed
COH: Controlled ovarian overstimulation
PDE3-I: Phosphodiesterase 3 inhibitor
ADSCs: Adipose-derived stem cells
collagen/UC-MSCs: Umbilical cord mesenchymal stem cells on collagen scaffolded
FOXO3a: Forkhead box O3
FOXO1: Forkhead box protein O1
HA: Hyaluronic acid
ALG: Alginate
HAA: A composite hydrogel composed of HA and ALG
COC: Cumulus oocyte complex
HABH: Hyaluronic acid-based hydrogel
VEGF: Vascular endothelial growth factor
ESC-MPCs: Embryonic stem cells-derived mesenchymal progenitor cells
TG: Tripterygium glycosides
PGRMC1: Progesterone receptor membrane component 1
FIB: Fibrin
FA: Fibrin-alginate
FA-IPN: Fibrin-sodium alginate interpenetration network
SCID: Severe combined immunodeficiency
F/T: Fibrinogen/thrombin
HBP: Heparin binding peptide
bFGF: Basic fibroblast growth factor
NO-NPs: Nitric oxide releasing nanoparticles
M: 1, 4-Bound B-d-mannuronic acid
G: A-l-guluronic acid
2D: Two-dimensional
BMP-4: Bone morphogenetic protein-4
PEG-VS: Poly (ethylene glycol) vinyl sulfone
RTK: Receptor tyrosine kinase
PTFE: Polytetrafluoroethylene
VHOS: Ovarian spherical vascularized hydrogels
OP: Osteoporosis
NPs: Nanoparticles
Fe3O4: Ferric oxide
OVX: Ovariectomized
CD-MBGNPs: A localized E2 delivery system with incorporation of β-cyclodextrin
AD: Alzheimer's disease
CNS: Central nervous system
pro-E2: A biomaterial scaffold composed of electrospinning fibers and films completely composed of poly
CVD: Cardiovascular disease
SUI: Stress urinary incontinence
CuNPs: Copper nanoparticles
